# An updated herpetological inventory of the Agusan Marsh Wildlife Sanctuary, eastern Mindanao Island, Philippines

**DOI:** 10.3897/zookeys.1272.167304

**Published:** 2026-03-09

**Authors:** Marites B. Sanguila, Ana Cristina O. Labaja, Justin M. Bernstein, Rafe M. Brown

**Affiliations:** 1 Biodiversity Informatics and Research Center, Father Saturnino Urios University, San Francisco St., Butuan City, Agusan del Norte, 8600, Philippines Biodiversity Institute and Department of Ecology and Evolutionary Biology, University of Kansas Lawrence United States of America; 2 Natural Sciences and Mathematics Division, Father Saturnino Urios University, San Francisco St., Butuan City, Agusan del Norte, 8600, Philippines Department of Mammalogy, Division of Vertebrate Zoology, American Museum of Natural History New York United States of America; 3 Department of Mammalogy, Division of Vertebrate Zoology, American Museum of Natural History, New York, New York 10024, USA Biodiversity Informatics and Research Center, Father Saturnino Urios University Butuan City Philippines; 4 Biodiversity Institute and Department of Ecology and Evolutionary Biology, University of Kansas, Lawrence, Kansas 66045, USA Natural Sciences and Mathematics Division, Father Saturnino Urios University Butuan City Philippines

**Keywords:** Biodiversity, checklist, conservation, freshwater swamp, management, peat swamp, wetland

## Abstract

A formal synthesis of the occurrence of herpetofauna species in Agusan Marsh Wildlife Sanctuary remained scant since the documentation of historical records by E.H. Taylor in the early 20^th^ century. Here, an updated checklist of the Agusan Marsh herpetofauna is provided. 49 species (16 amphibians and 33 reptiles) are recorded from the Agusan Marsh’s peat and freshwater swamp forests, specifically, amphibians in the families Bufonidae, Ceratobatrachidae, Dicroglossidae, Microhylidae, Megophryidae, Ranidae, and Rhacophoridae; lizards in the families Agamidae, Gekkonidae, Dibamidae, Scincidae, and Varanidae; snakes in the families Colubridae, Cyclocoridae, Psammodynastidae, Pareidae, Pythonidae, Typhlopidae, and Viperidae; and a turtle in the family Geoemydidae. Our Shannon Diversity Indices calculations suggest that diversity might be declining, but more surveys are needed to sample the total species richness of the Agusan Marsh Wildlife Sanctuary. Our checklist highlights 14 new records (6 amphibians, 8 reptiles) and the presence of four invasive alien species in Agusan Marsh. Given the tremendous historical significance of the region, Agusan Marsh may represent one of the most significant focal study sites for assessing the impacts of historical land use and climate change in the Philippines. This work also demonstrates the importance of temporally sequential survey-resurveys for updating baseline biodiversity data to inform management decisions and conservation actions for this unique wetland ecosystem in Southeast Asia.

## Introduction

An extensive list of herpetological species occurrences (20 amphibians, 52 reptiles) from the Agusan River Basin can be gleaned from the monographs by E. H. Taylor, who recorded observations during his travels through Butuan, Talacogon, the Upper Agusan Valley (Taylor 1915, 1917a, b, 1918a, b, 1919, 1920a, b, 1921, 1922a, 1922b, 1922c, 1922d, 1922e, 1923, 1925, 1928, 1960, 1963a, b, 1965), and his extended stay in Bunawan during the years of 1912 and 1913 (Taylor 1975). Taylor’s work in the Agusan River Basin is essentially the only long-term, longitudinal account of unique historical records of herpetofauna from a single general area in the archipelago (Inger 1954, 2008). Taylor’s surveyed sites (see Taylor 1975) are now enclosed within the formally designated Agusan Marsh Wildlife Sanctuary (AMWS) or simply Agusan Marsh.

Recent herpetological surveys in the Agusan Marsh have been focused on just a few relatively accessible sites (Ibañez and Bastian 2004; Almeria and Nuñeza 2013; Ngilangil et al. 2014; Sularte et al. 2015; Relox and Camino 2018; Gamalinda et al. 2024), have not involved regular sampling periods, have consisted of brief and variable durations, and have considered little to no spatial, temporal, or seasonal environmental variation, leaving large portions—including most of the interior of the overall wetland—unstudied. The absence of a deliberately structured, repeated, or temporally sequential approach (Morrison et al. 2008; Pereira et al. 2017) has resulted in a shortfall of biodiversity knowledge (Silveira et al. 2010) pertaining to the Agusan Marsh fauna (e.g., Alviola et al. 2023).

The shortfall represented by the historically incomplete knowledge of the herpetofauna of Agusan Marsh and adjacent surrounding areas is likely attributable to factors such as logistical challenges to fieldwork, inadequate funding (limiting the number of technical personnel for the park), budgetary shortages limiting equipment and supplies, or underdeveloped capacity, limited opportunities for training of local stakeholders, and the remote and inhospitable landscape itself (Gibbens 2021; Wetlands Link International 2025). These factors combine to represent a challenge for natural resource management of the protected area that disrupts strategic planning and mitigation of problems, and all but prevents proactive sustainable management (Brito 2010; Brown et al. 2012a; Hortal et al. 2015; Oliveira et al. 2016; Vergara-Asenjo et al. 2023).

**Figure 1.**
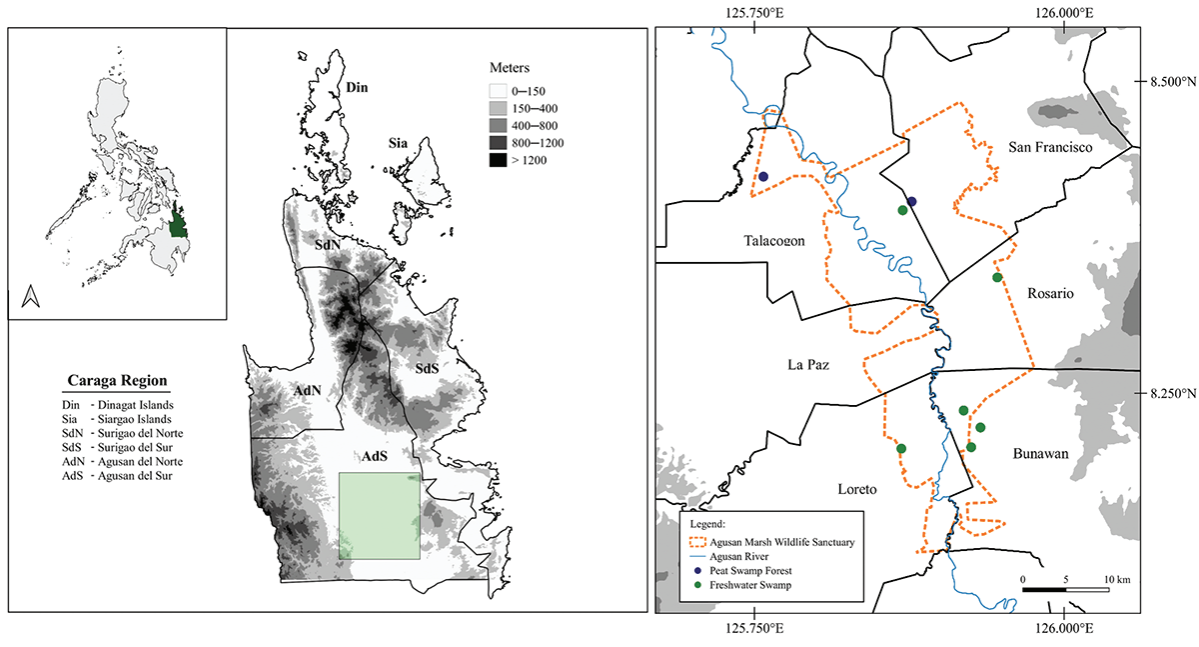
Map of the Philippines depicting northeast Mindanao and the sampling sites (green shade; see inset) in Agusan Marsh Wildlife Sanctuary.

The Agusan Marsh is composed of a complex ecological network or mosaic of unique habitats, including peatland and mixed swamp forests, marshes, ponds, and lakes—all of which are seasonally connected-and-disconnected by intermittent streams and river drainages (Taylor 1975; Makinano-Santillan and Santillan 2021). These unique habitats provide ecological services for the densely surrounding human population (Fig. 1), serving the surrounding communities of the entire Caraga Region: the Provinces of Agusan del Norte, Agusan del Sur, Surigao del Norte, and Surigao del Sur; and all the way south to the Davao City areas. These ecosystems function as sources of high-quality fresh water for human consumption, irrigation for crops, as a natural surface reservoir system for the provision of water during annual dry periods, and as a catch basin for flood control (Mora-Garcia et al. 2020; Key Biodiversity Areas Partnership 2025). Additionally, for the immediately surrounding communities, the wetlands directly provide livelihood, and a primary local source of fish and other renewable foods (Baclayo et al. 2020; Campos Jr et al. 2023).

During the last century, the Caraga Region human population has greatly expanded (Commission on Population and Development 2025), while the majority of Mindanao Island’s low-elevation dipterocarp and hardwood closed-canopy forests have been systematically removed (Kummer 1992; Vitug 1996; Chokkalingam et al. 2006), including the dense forests that previously surrounded the Agusan Marsh wetland (see Taylor 1975). This has resulted in dense human population encroachment and development, almost to the edge of the Agusan Marsh wetland, on all sides of the marsh (Asian Development Bank 2008; Varela et al. 2013; Sespeñe et al. 2016; Gibbens 2021). Because human population pressure is so intense, surrounding the entire wetland, and continues to encroach into the delicate protected wetland itself (with a few, patchily distributed and non-continuous ‘buffer zones’ separating developed areas from the marsh itself), the potential for continued degradation of wetland habitats is considerable. Additionally, these factors combine to increase the likelihood of human–wildlife interactions, incursions into the protected area, poaching, and/or consumptive over-harvesting of the wetland’s biodiversity and renewable resources (Varela et al. 2013; Sespeñe et al. 2016; Baclayo et al. 2020; Paz and Gonzales 2021). Because these threats are ongoing, repeated surveys will be necessary to assess the effects and identify sources of species introductions (Diesmos et al. 2006; Pili et al. 2019; Que et al. 2020), consider the impacts associated with exploitation or unsustainable harvest of marsh renewable resources, evaluate the effects of water diverted for irrigation (Tomas et al. 2011; Gibbens 2021), monitor status of edge habitats, and build a longitudinal dataset (Brown et al. 2012a; Alviola et al. 2023), capable of informing land managers (via rigorous scientific baseline data and simple, transparent, reproducible methods) and empowering stakeholders to mitigate threats (Mora-Garcia et al. 2020; Davies et al. 2020; Campos Jr et al. 2023). Maintaining the integrity, ecological function, diversity of habitat types, and the variable biological communities they each support (Mallari et al. 2001; Sespeñe et al. 2016; Gibbens 2021) will likely require regularly scheduled, vigilantly deployed, and scientifically empowered (transparent, reproducible, and defensible; employing simple but rigorous methods) follow-up surveys and re-surveys.

Here we document recent occurrences of AMWS amphibian and reptile species, to ameliorate the historical hiatus of the last century, provide an urgently needed update to the known herpetofaunal species richness, and demonstrate the feasibility of continued, sustained, survey-and-resurvey efforts. We take a first step towards the goal of capacitating regular, periodic, future herpetological assessments of the Agusan wetlands by synthesizing results of recent field surveys and new data collected by us, for comparison to the historical faunal inventory provided by Taylor (1920a, 1922a-e, 1975).

We document the continued persistence of apparently large, robust populations of species unrecorded since their original discovery in the early 1900s, but which now may be threatened by the activities of humans surrounding the marsh. We also report on the alarming presence of invasive species that have colonized the area over the last century or have been introduced—clearly, since Taylor’s work (Taylor 1920a, 1922a, 1975). Given the unique century-long, before-and-after comparison provided by the opportunity to revisit Taylor’s studies, we call for an exhaustive, continued, seasonally variable, and taxonomically comprehensive herpetological inventory of the wetland, which will be required to inform management decisions and guide biologically sound conservation action necessary to protect the unique and threatened Agusan Marsh Wildlife Sanctuary.

## Materials and methods

### Study area

The Agusan Marsh Wildlife Sanctuary (**AMWS**; Fig. 1) is a protected area under the Philippine Republic Act 7586 (National Integrated Protected Areas System Law; NIPAS) by virtue of Presidential Proclamation 913, issued on 31 October 1996. The wetland is also designated as an Important Birdlife Area (IBA: assessed in 2001), a Ramsar Site (Convention on Wetlands 2025: 12 November 1999), the ninth ASEAN Heritage Park in the Philippines (ASEAN Center for Biodiversity 2018), and together have the distinction of being the second largest wetland in the Philippines (Sespeñe et al. 2016). Agusan Marsh is the only freshwater ecosystem among the nine key biodiversity areas of the Caraga Region, constituting the northernmost reaches of the Eastern Mindanao Biodiversity Corridor (Philippine Eagle Foundation 2015; BirdLife International 2025; Convention on Wetlands 2025). It is also currently on the tentative list for the UNESCO Heritage Site (UNESCO 2025).

The Agusan Marsh protected area covers 191.97 km^2^, with an elevation of 16–21 meters (Sumilhig et al. 2024), encompassing six municipalities (Bunawan, Rosario, Loreto, La Paz, Talacogon, and San Francisco) in the Province of Agusan del Sur. Similar to other protected areas in the country, the AMWS is managed by the Protected Area Superintendent Office (PASu; now Protected Area Management Office; PAMO) and consists of three categories of distinctly defined, ‘delineated zones’ (Laviña et al. 2010). These include multiple-use zones (for settlement and land use), nine strict protection zones (areas of high biodiversity value, close to human settlements, for research use only), and buffer zones (areas outside the protected zone; Laviña et al. 2010).

Geologically, the west and north portions of Agusan Marsh (municipalities of Loreto, La Paz, Talacogon, and San Francisco) are characterized by Pliocene-Pleistocene deposits, predominantly containing alluvium soils (unpublished data, AMWS Management Plan 2015–2020; on file in the office of the protected area Superintendent [pers. comm. PASu Emmillie Iboña to MBS, 5 December 2018).

Under the Modified Coronas Classification (MCC), the climate of Agusan Marsh is characterized by a Type II designation (no dry season, pronounced rainy periods, from December to February), with a Type IV (rainfall evenly distributed throughout the year). The marsh has an average monthly rainfall of 355 mm, and a mean temperature of 27.15 °C (unpublished data, AMWS Management Plan 2015–2020; on file in the office of the protected area Superintendent [pers. comm. PASu Emmillie Iboña to MBS, 5 December 2018).

In the peatlands of Agusan Marsh, vascular plant communities are dominated by *Tristaniopsis decorticata* and *Thoracostachyum sumatranum* (Aribal and Fernando 2018). In the forested marsh areas, important waterfowl, such as herons and egrets (Mallari et al. 2001; BirdLife International 2025), and ~124–150 other species of birds have been documented (Sucaldito-Salibad and Nuñeza 2014; Sumilhig et al. 2024). Fish communities in the floodplain lakes of Agusan Marsh are severely altered by the activities of humans, subsistence fish farming, and the deliberate introductions (stocking) of non-native food fish; when last studied, the numbers of introduced fish species exceeded the numbers of native species (Jumawan and Seronay 2017).

### Collection and field methods

**Figure 2.**
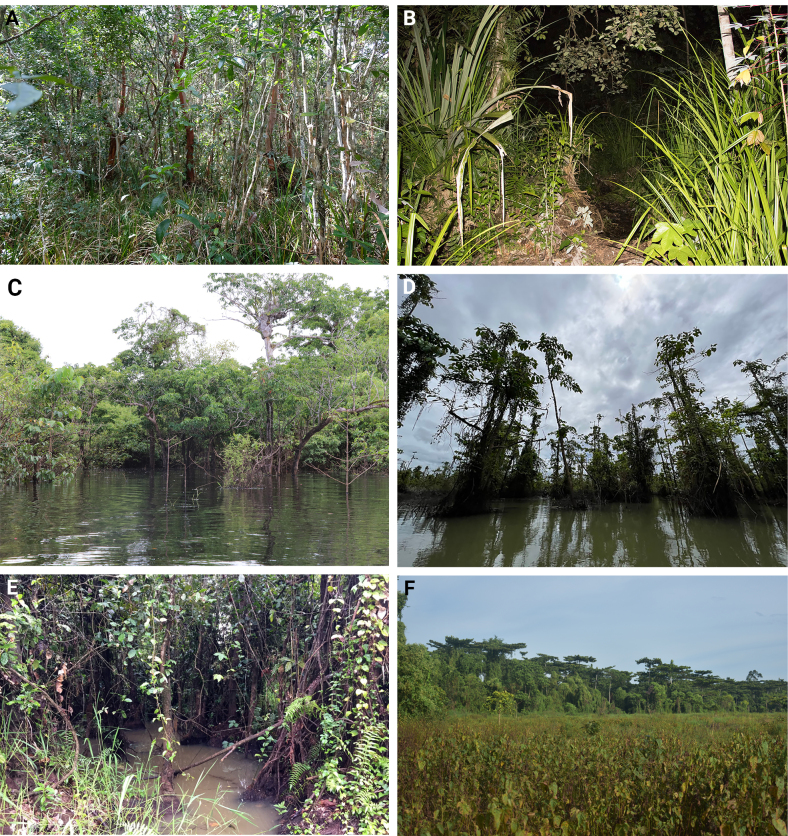
Swamp habitats in Agusan Marsh Wildlife Sanctuary. **A**. Caimpugan Peatland; **B**. Katigbok peat swamp; **C**. Lake Kasawangan, freshwater swamp; **D**. Lake Tugakon, freshwater swamp; **E**. Mixed swamp forest, disturbed; **F**. Mixed swamp forest with *Terminalia* trees.

We conducted fieldwork within the municipalities of Bunawan, Rosario, San Francisco, Talacogon, and Loreto. Peat swamp forests were surveyed from 28 April–13 May 2008; from 11–13 September 2019, and 14–16 March 2022 in Caimpugan, San Francisco; and from 28–30 January 2022 in Katigbok, Talacogon. From 2–5 December 2019, we surveyed mixed peat swamp forest dominated by *Terminalia* trees, and in open riverbank habitats and agricultural areas in the Municipality of Rosario. Surveys were also conducted between 28 April and 13 May 2008, 13–15 December 2019, and 8–11 April 2021 along a riverbank habitat and mixed swamp forest in the Municipality of Bunawan, and from 18–24 April 2022 and 26–28 April 2022 in freshwater swamp forest in the Municipalities of Talacogon and Loreto (Figs 1, 2a–f).

Visual encounter surveys were undertaken by experienced field workers to identify and record species occurrences. Specimens were photographed in life, their microhabitats recorded, and their positions georeferenced. When vouchers were required to confirm identification, specimens were euthanized using a 2% Lidocaine solution. We dissected and euthanized specimens in the field to remove liver tissue from vouchers, and we preserved these genetic samples separately in 100% laboratory-grade ethanol. Whole-body voucher specimens were then preserved by injection and fixation with 10% buffered formalin. Following fixation in formalin (≤ 2 weeks), specimens were rinsed with water, transferred in 70% ethanol, and permanently deposited at two biodiversity repositories, to ensure safety/assurance (via physical and locational redundancy) of the collection, and to observe equitable sharing of derived resources; data security was achieved by instantaneous OpenAccess web-based serving of all specimen-associated data, following DarwinCore standards (Wieczorek et al. 2012; Hedrick et al. 2020; Hardisty et al. 2022).

Specimens of most species are currently housed at the FSUU-BIRC Natural History Collection (https://scientific-collections.gbif.org/institution/da4bad98-f46a-4ec2-8b9d-e6e8d7ac786f; cataloged specimen codes BIRC, MBS) and the KU Herpetology Collections (https://www.gbif.org/publisher/b554c320-0560-11d8-b851-b8a03c50a862; cataloged specimen code KU). Specimens were collected under Gratuitous Permits (GPs) Nos. R13-2019-55, R13-2019-56, Renewal R13-2021-02 issued by the Philippine Department of Environment and Natural Resources–Caraga Regional Office to MBS, and GP 185 (Renewal) issued to RMB by the Protected Areas and Wildlife Bureau (now Biodiversity and Management Bureau) of the Philippine Department of Environment and Natural Resources-Central Office (Quezon City, Manila). We processed and published our dataset online in the Global Biodiversity Information Facility (https://doi.org/10.15468/t9a5uj).

Vouchers were examined to confirm morphological character states associated with species diagnoses, using a stereomicroscope (Leica S9D, EZ4 W), appropriately guided by available taxonomic keys and/or primary literature (see species identification in Results, below) by Taylor (1920a; 1922a, b, c, e), Alcala and Brown (1998, 1999), Brown (1991), Brown and Alcala (1978, 1980, 1994), Linkem et al. (2010, 2011), Brown et al. (2012b), Diesmos et al. (2015), Sanguila et al. (2016), Barley et al. (2020), Leviton et al. (2018), and Weinell et al. (2019). We resolved taxonomic identities of all species recorded from historical and recent surveys (Taylor 1920a, 1922a; Almeria and Nuñeza 2013; Ngilangil et al. 2014; Ibañez and Bastian 2004; Sularte et al. 2015; Relox and Camino 2018). We followed amphibian nomenclature in the Amphibian Species of the World database (Frost 2025) and AmphibiaWeb taxonomic subcommittee (AmphibiaWeb 2025), and reptile nomenclature followed the usage of The Reptile Database (Uetz et al. 2025).

To determine how well our survey efforts were at sampling the overall biodiversity of the AMWS, we created species accumulation curves using a custom script in R v4.1.2 using the packages *dplyr* v1.1.2 (Wickham et al. 2023), *tidyr* v1.3.0 (Wickham et al. 2024), and *vegan* v2.5-7 (Oksanen et al. 2025). We calculated species accumulation curves for all habitats combined, as well as for each habitat type individually (peat swamps, mixed swamps, and freshwater swamps). For each species accumulation curve, we calculated the mean Shannon Index (*H_x̄_*) (https://github.com/jbernst/AgusanMarsh_Survey; https://doi.org/10.5281/zenodo.15580934).

## Results

**Figure 3.**
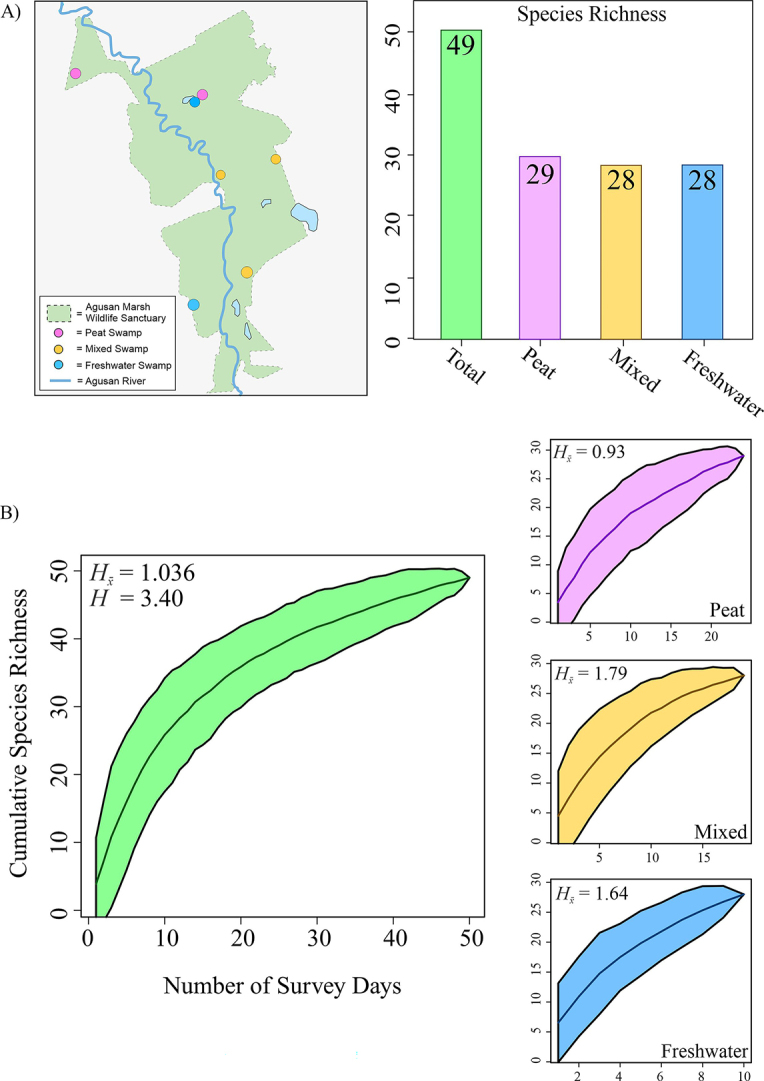
Herpetological species diversity in Agusan Marsh Wildlife Sanctuary. **A**. Bar plots show total species richness for combined and individually sampled habitats; **B**. SACS: Species Accumulation Curves for categories in Panel A, with median cumulative species richness, 95% confidence intervals. Shannon Diversity Index (*H*) and mean (*H_x̄_*) are provided.

By not yet approaching asymptotes, the species accumulation curves (for all habitats combined and habitats individually) indicate that more surveys are needed to sample the total biodiversity of the Agusan Marsh Wildlife Sanctuary (Fig. 3). The total species richness for total (cumulative – all habitats), peat swamp, mixed swamp, and freshwater swamps was 49, 29, 28, and 28, respectively (Fig. 3A). Although the species richness was 49 for the entire survey period across all years, the species richness for all habitats when only using data from 2019, 2021, and 2022 was 42. Mixed swamp habitats had the highest mean Shannon Diversity Index (*H_x̄_* = 1.79), followed by freshwater swamps (*H_x̄_* = 1.64), total (all habitats; *H_x̄_* = 1.04), and peat swamps (*H_x̄_* = 0.93). When all habitats were combined across all years, the Shannon Diversity Index (H) was 3.40 (Fig. 3B).

Table 1 summarizes our recent confirmations of species identities (49 species; 16 amphibians and 33 reptiles) within the broader geographic context of the herpetofauna of the AMWS. Below, we present species accounts for taxa recorded from our (2008 and 2019–2022) field surveys and comment on novel findings that contribute new information to our cumulative knowledge of the herpetofauna of Agusan Marsh. We present information on the sex of the individual(s) when available.

**Table 1.** Species list of the herpetofauna in Agusan Marsh Wildlife Sanctuary. PS (Peat Swamp), FS (Freshwater Swamp, including Mixed Swamp), OS (Other Surveys), HR (Historical Record).

Order	Family	Scientific Name	Present Survey		Other Sources	
			PS	FS	OS	HR
Anura	Bufonidae	*Ansonia muelleri*			X	
		*Rhinella marina*		X	X	
		*Pelophryne lighti*			X	X
	Ceratobatrachidae	*Platymantis corrugatus*	X			
		*Platymantis guentheri*	X			
	Dicroglossidae	*Fejervarya moodiei*				X
		*Fejervarya vittigera*	X	X	X	
		*Hoplobatrachus rugulosus*		X	X	
		*Limnonectes leytensis*	X	X	X	X
		*Limnonectes magnus*			X	
		*Limnonectes parvus*				X
		*Occidozyga laevis*			X	X
	Microhylidae	*Chaperina fusca*			X	X
		*Kalophrynus sinensis*	X		X	X
		*Kaloula conjuncta*				X
		*Kaloula picta*		X		
		*Kaloula pulchra*		X		
		*Oreophryne anulata*	X	X	X	X
	Megophryidae	*Leptobrachium lumadorum*			X	
		*Megophrys stejnegeri*			X	X
	Ranidae	*Hylarana grandocula*			X	X
		*Staurois natator*			X	X
		*Indosylvirana nicobariensis*	X	X		
	Rhacophoridae	*Kurixalus appendiculatus*	X	X		
		*Philautus acutirostris*	X		X	X
		*Philautus leitensis*	X			
		*Polypedates leucomystax*	X	X	X	X
		*Rhacophorus pardalis*				X
		*Rhacophorus bimaculatus*				X
		*Nyctixalus spinosus*	X			X
Squamata	Agamidae	*Draco bimaculatus*	X			X
		*Draco ornatus*				X
		*Gonocephalus semperi*	X		X	
		*Hydrosaurus pustulatus*		X		X
	Gekkonidae	*Cyrtodactylus agusanensis*			X	X
		*Cyrtodactylus annulatus*	X	X		X
		*Gehyra mutilata*		X		X
		*Gekko monarchus*				X
		*Hemidactylus frenatus*		X		X
		*Hemiphyllodactylus typus*	X	X		
		*Lepidodactylus aureolineatus*		X		X
		*Gekko intermedium*				X
	Dibamidae	*Dibamus leucurus*				X
		*Brachymeles orientalis*	X			
		*Brachymeles schadenbergi*				X
		*Emoia ruficauda*	X	X		X
		*Eutropis caraga*		X		
		*Eutropis cumingi*				X
		*Eutropis lapulapu*	X			
		*Eutropis multifasciata*	X	X	X	
Squamata	Dibamidae	*Dasia semicincta*				X
		*Lamprolepis smaragdina*		X		
		*Tropidophorus misaminius*			X	X
		*Lipinia pulchella*		X		X
		*Lipinia quadrivittata*	X	X		X
		*Lipinia semperi*		X		X
	Scincidae	*Parvoscincus steerei*	X			
		*Pinoyscincus jagori*		X	X	X
		*Pinoyscincus mindanensis*				X
		*Sphenomorphus diwata*			X	
		*Sphenomorphus fasciatus*		X		X
		*Sphenomorphus variegatus*			X	
	Varanidae	*Varanus cumingi*		X		X
	Colubridae	*Ahaetulla prasina*				X
		*Boiga dendrophila latifasciata*		X		X
		*Chrysopelea paradisi*		X		X
		*Coelognathus erythrurus erythrurus*		X		X
		*Dendrelaphis marenae*		X		
		*Dendrelaphis philippinensis*		X		
		*Oligodon maculatus*				X
	Cyclocoridae	*Oxyrhabdium modestum*	X	X		X
	Elapidae	*Calliophis philippina*				X
		*Naja samarensis*		X	X	X
	Natricidae	*Rhabdophis auriculata*			X	X
		*Rhabdophis lineatus*		X		
	Psammodynastidae	*Psammodynastes pulverulentus*	X		X	X
	Pareidae	*Aplopeltura boa*	X			X
	Pythonidae	*Malayopython reticulatus*			X	X
	Typhlopidae	*Ramphotyphlops cumingii*				X
	Viperidae	*Trimeresurus flavomaculatus*			X	X
		*Tropidolaemus subannulatus*	X			X
	Geoemydidae	*Cuora philippinensis*		X		X

### ANURA Duméril, 1806

#### Frogs and Toads

**Family Bufonidae Gray, 1825**

##### Rhinella marina

Taxon classificationAnimaliaAnuraBufonidae

(Linnaeus, 1758)

7D6631E0-99F5-5B1A-9EEF-C2FDC18E184F

Fig. 4A

###### Vernacular name.

American Toad

###### Material examined.

Philippines • 1 individual; Mindanao, Agusan del Sur Province, Municipality of Rosario, Barangay Novele, Sitio Palibo; 08°20'34"N, 125°56'46"E; BIRC 761 • 1 individual; Municipality of Bunawan, Sitio Mambalili; 08°12'24"N, 125°55'30"E; BIRC 602 • 1 individual; Municipality of Talacogon, Lake Kasawangan, Barangay La Flora; 08°23'48"N, 125°52'11"E; MBS 2160.

**Figure 4.**
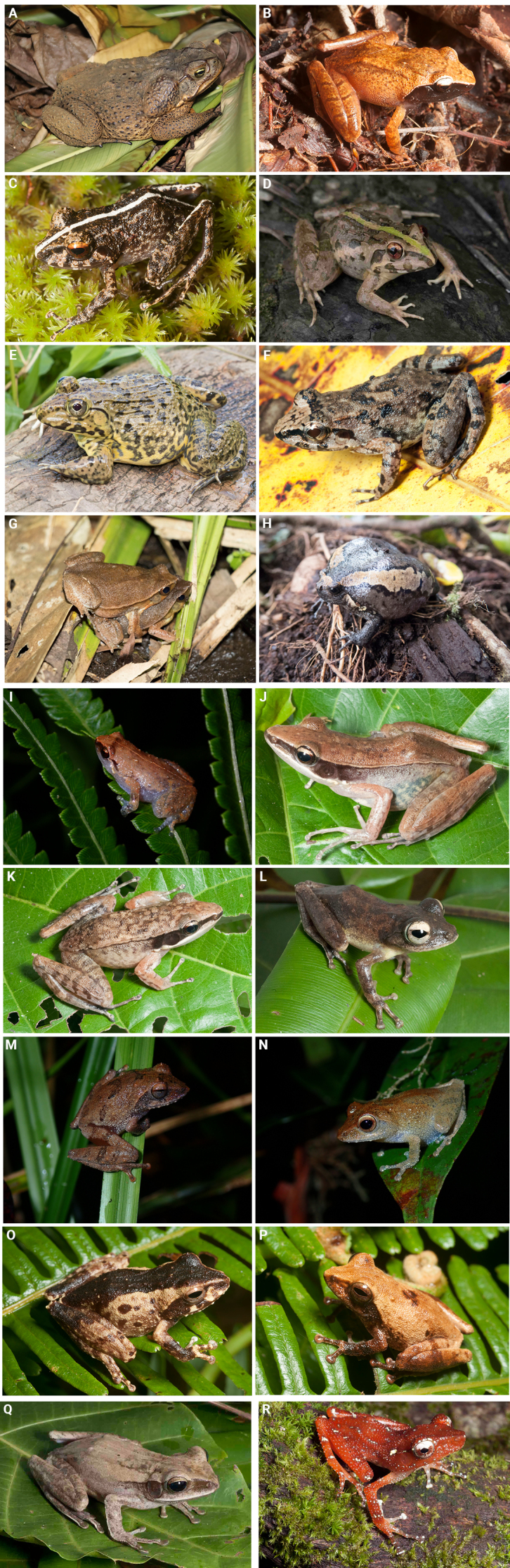
Amphibians recorded from Agusan Marsh Wildlife Sanctuary. **A**. *Rhinella marina*; **B**. *Platymantis corrugatus*; **C**. *Platymantis guentheri*; **D**. *Fejervarya vittigera*; **E**. *Hoplobatrachus rugulosus*; **F**. *Limnonectes leytensis*; **G**. *Kalophrynus sinensis*; **H**. *Kaloula pulchra*; **I**. *Oreophryne anulata*; **J**. *Indosylvirana nicobariensis*, female; **K**. *Indosylvirana nicobariensis*, male; **L**. *Kurixalus appendiculatus*; **M**. *Philautus leitensis*, color morph 1; **N**. *Philautus leitensis*, color morph 2; **O**. *Philautus leitensis*, color morph 3; **P**. *Philautus leitensis*, color morph 4; **Q**. *Polypedates leucomystax*; **R**. *Nyctixalus spinosus*.

###### Identification.

The combination of a brown, tuberculate, and leathery dorsum, a dirty cream venter with dark blotches, and a pair of parotoid glands located behind the eyes.

###### Remarks.

This invasive alien species occurs in dense aggregations, typically numbering quite a few (>10) individuals, in agricultural areas surrounding the peat swamp forest and in freshwater swamps with mixed vegetation of shrubs, *Terminalia*, and other marsh-dwelling trees.

**Family Ceratobatrachidae Boulenger, 1884**

##### Platymantis corrugatus

Taxon classificationAnimaliaAnuraCeratobatrachidae

(Duméril, 1853)

1CFCAB1F-87EE-5B5D-82C0-04506713AE29

Fig. 4B

###### Vernacular name.

Corrugated Tagomukhus Forest Frog

###### Material examined.

Philippines • 3 individuals; Mindanao, Agusan del Sur Province, Municipality of San Francisco, Barangay Caimpugan; 08°24'12"N, 125°53'49"E; KU 314062–64 • 10 individuals; same locality data as for preceding; 08°24'13"N, 125°52'37"E; MBS 1967–68, 1974–76, 1989–93 • 2 individuals; Municipality of Talacogon, Barangay Zillovia; 08°25'25"N, 125°45'26"E; MBS 1742, 1752.

###### Identification.

A gray, tan, or brown dorsum interspersed with irregular longitudinal folds; most individuals possess distinct dark “face masks” with dark pigmentation from the nares, through the loreal region, the pre- and post-ocular region, to the tympanic region (Taylor 1920a; Inger 1954).

###### Remarks.

Individuals of this crepuscular species were heard actively calling, hiding underneath leaf litter on the forest floor in the Katigbok and Caimpugan peat swamp forests of Agusan Marsh. It occurs throughout the Philippine archipelago except in Palawan and Sulu PAICs (Diesmos et al. 2015). Populations in the Mindanao faunal region often possess a less darkly pigmented lateral head surface (Alcala and Brown 1998, 1999; Brown et al. 2012b; Diesmos et al. 2015; Cobb 2016).

##### Platymantis guentheri

Taxon classificationAnimaliaAnuraCeratobatrachidae

(Boulenger, 1882)

664C56BE-0635-5E1C-BEC3-E7647A78CE0E

Fig. 4C

###### Vernacular name.

Understory Tahananpuno Rainfrog

###### Material examined.

Philippines • 4 individuals; Mindanao, Agusan del Sur Province, Municipality of San Francisco, Barangay Caimpugan; 08°13'21"N, 125°55'57"E; MBS 1966, 1983, same locality data as for preceding; 08°24'13"N, 125°52'37"E; MBS 1986, 1994 • 3 individuals; Municipality of Talacogon, Barangay Zillovia; 08°25'25"N, 125°45'26"E; MBS 1749–50, 1774.

###### Identification.

A visible tympanum, a prominent series of irregularly variable tubercular ridges on its dorsum, and expanded terminal digits of fingers and toes (Alcala and Brown 1998, 1999; Diesmos et al. 2015).

###### Remarks.

This species actively calls while perched 1–3 m high, in shrubs or on branches or leaves of understory trees, often covered with moss, in peat swamp forests of Agusan Marsh.

**Family Dicroglossidae Anderson, 1871**

##### Fejervarya vittigera

Taxon classificationAnimaliaAnuraDicroglossidae

(Weigmann, 1824)

B1DFECBA-ADD0-5047-A6B1-CFEC4A4C33BE

Fig. 4D

###### Vernacular name.

Philippine Pond Frog

###### Material examined.

Philippines • 3 individuals; Mindanao, Agusan del Sur Province, Municipality of Bunawan, Barangay San Marcos; confluence of Simulao and Agusan Rivers; 08°13'21"N, 125°55'57"E; KU 314051–53; 4 individuals; same locality data as for preceding; Sitio Mambalili; 08°12'24"N, 125°55'30"E; BIRC 834, 840–42 • 6 individuals; same locality data as for preceding; 08°12'24"N, 125°55'30"E; BIRC 599–601, 627–29 • 7 individuals; Municipality of Loreto, Barangay Poblacion; 08°12'20"N, 125°52'07"E; MBS 2232–35, 2237–39.

###### Identification.

An olive gray dorsum, a pale vertebral line from snout to cloaca occasionally present (absent in most individuals), visible bright yellow bars on hindlimbs or groin region surfaces, dorsal surface of digits smooth, and the absence (vs present in *F. moodiei*) of a flap of skin on the outer surface of the tarsus and outer (fifth) toe (Taylor 1920a; Inger 1954; Alcala and Brown 1998; Diesmos et al. 2015).

###### Remarks.

In Agusan Marsh, individuals occur in and along irrigation channels of rice fields, and in quiet pools of water adjacent to small creeks and rivers.

##### Hoplobatrachus rugulosus

Taxon classificationAnimaliaAnuraDicroglossidae

(Osbeck, 1765)

D7469D7B-76C9-5DCB-8EC0-2ECE81369872

Fig. 4E

###### Vernacular name.

Taiwanese Bullfrog

###### Material examined.

Philippines • 1 individual; Mindanao, Agusan del Sur Province, Municipality of Talacogon, Barangay Zillovia; 08°25'25"N, 125°45'26"E; MBS 1461.

###### Identification.

A stocky body and dark yellowish-brown dorsum covered with numerous short ridges (Diesmos et al. 2006, 2015).

###### Remarks.

Like other invasive alien species, *H. rugulosus* occurs in areas converted for agriculture, at sites surrounding peat swamp forest, and freshwater swamps of Agusan Marsh.

##### Limnonectes leytensis

Taxon classificationAnimaliaAnuraDicroglossidae

(Boettger, 1893)

65A2753E-A284-5D38-8CB8-44185BE1E8CB

Fig. 4F

###### Vernacular name.

Leyte Fanged Frog

###### Material examined.

Philippines • 7 individuals; Mindanao, Agusan del Sur Province, Municipality of Rosario, Barangay Novele; Sitio Palibo; 08°20'34"N, 125°56'46"E; BIRC 807–13 • 3 individuals; Municipality of Bunawan, Barangay San Marcos; confluence of Simulao and Agusan Rivers; 08°14'10"N, 125°55'08"E; KU 314059–61 • 11 individuals; same locality data as for preceding; 08°14'10"N, 125°55'08"E; BIRC 611, 630, 632–34, 836, 854–58 • 4 individuals; Municipality of Talacogon, Barangay Zillovia; 08°25'25"N, 125°45'26"E; MBS 1753, 1771–73 • 2 individuals; Municipality of Loreto, Barangay Poblacion; 08°12'20"N, 125°52'07"E; MBS 2220, 2240.

###### Identification.

A dark olive brown or gray dorsum, a distinct inverted V-shaped fold in the scapular region, a bar of dark pigmentation spanning the space between the upper eyelids, dorsal surface of digits with a groove/fold, and dark transverse crossbars on hindlimbs (Taylor 1920a; Inger 1954; Alcala and Brown 1998; Diesmos et al. 2015).

###### Remarks.

In Agusan Marsh, individuals occur in converted and abandoned agricultural areas contiguous with peat swamps with *Terminalia* trees and in freshwater swamps adjacent to rivers and creeks.

**Family Microhylidae Günther, 1858**

##### Kalophrynus sinensis

Taxon classificationAnimaliaAnuraMicrohylidae

Peters, 1867

D119AECB-0BF1-5F18-97D4-DBB70198EC88

Fig. 4G

###### Vernacular name.

Philippine Sticky Frog

###### Material examined.

Philippines • 2 individuals; Mindanao, Agusan del Sur Province, Municipality of San Francisco, Barangay Caimpugan; 08°24'13"N, 125°52'37"E; KU 314054–55 • 1 ♂, 1 ♀; Municipality of Talacogon, Barangay Zillovia; 08°25'25"N, 125°45'26"E; MBS 1775–76.

###### Identification.

A creamy, brownish dorsum, possessing a dark X-shaped color pattern from each eyelid to the opposite groin region, two dark circular inguinal spots present, and a tan to pink venter with scattered dark pigmentation (Taylor 1920a; Inger 1954; Alcala and Brown 1998; Diesmos et al. 2015).

###### Remarks.

This species occurs in the Katigbok and Caimpugan peat swamp forests of Agusan Marsh.

##### Kaloula picta

Taxon classificationAnimaliaAnuraMicrohylidae

(Duméril & Bibron, 1841)

44EBEE54-414F-5DFF-A18F-701745A63C9D

###### Vernacular name.

Philippine Narrowmouth Toad

###### Material examined.

Philippines – Mindanao, Agusan del Sur Province • Municipality of Bunawan, Barangay San Marcos; 08°13'21"N, 125°55'57"E; KU 314056–57.

###### Identification.

An olive brown dorsum, possessing a distinct, large, irregular, variable dark medial blotch or irregular, elongate marking, a pale brown to pinkish-tan venter, and webbed toes with non-expanded to only slightly expanded terminal discs (Taylor 1920a; Inger 1954; Alcala and Brown 1998; Diesmos et al. 2015).

###### Remarks.

This species occurs in areas adjacent to open river systems in the swamps of Agusan Marsh.

##### Kaloula pulchra

Taxon classificationAnimaliaAnuraMicrohylidae

Gray, 1831

CA423D71-057D-5277-ADE3-8F0936B4566B

Fig. 4H

###### Vernacular name.

Malaysian Narrowmouth Toad

###### Material examined.

Philippines • 1 individual; Mindanao, Agusan del Sur Province, Municipality of Bunawan, Barangay San Marcos; confluence of Simulao and Agusan Rivers; 08°14'10"N, 125°55'08"E; BIRC 831 • 1 individual; Municipality of Talacogon, Lake Kasawangan, Barangay La Flora; 08°23'48"N, 125°52'11"E; MBS 2159.

###### Identification.

A variable, dark brown dorsum with darker and paler areas, beige-colored dorsolateral bands on each side of the body extending from behind the eye to the inguinal area, and a mottled venter (Sengupta et al. 2009; Diesmos et al. 2015).

###### Remarks.

In Agusan Marsh, individuals of this species occur in agricultural areas adjacent to peat swamp forest and freshwater swamps. This invasive alien species is widespread in the Philippines (Diesmos et al. 2015), with an expanding population trend (Diesmos et al. 2006; Pili et al. 2019).

##### Oreophryne anulata

Taxon classificationAnimaliaAnuraMicrohylidae

(Stejneger, 1908)

4968306B-6BD9-5641-8E66-76A4C66E028C

Fig. 4I

###### Vernacular name.

Mindanao Cross Frog

###### Material examined.

Philippines • 13 individuals; Mindanao, Agusan del Sur Province, Municipality of Rosario, Barangay Novele, Sitio Palibo; 08°20'34"N, 125°56'46"E; BIRC 569–75, 578–83 • 10 individuals; Municipality of Bunawan, Barangay San Marcos; confluence of Simulao and Agusan Rivers; 08°14'10"N, 125°55'08"E; BIRC 616–25, • 3 individuals; Municipality of Talacogon, Barangay Zillovia; 08°25'25"N, 125°45'26"E; MBS 1743–44, 1754 • 6 individuals; Municipality of Talacogon, Lake Kasawangan, Barangay La Flora; 08°23'48"N, 125°52'11"E; MBS 2136–41 • 3 individuals; Municipality of Loreto, Barangay Poblacion; 08°12'20"N, 125°52'07"E; MBS 2216, 2242, 2281.

###### Identification.

A stocky body, pale to dark brown dorsum with few irregular tubercles (absent in some individuals), mottled venter, and tips of fingers and toes expanded, with circummarginal grooves (Inger 1954; Alcala and Brown 1998; Diesmos et al. 2015).

###### Remarks.

This species appears to be common in the peat swamp forest and freshwater swamps (actively calling from floating vegetation) of Agusan Marsh.

**Family Ranidae Batsch, 1796**

##### Indosylvirana nicobariensis

Taxon classificationAnimaliaAnuraRanidae

(Stoliczka, 1870)

06EB928D-8C72-5220-A673-5915DC5CF739

Fig. 4J, K

###### Vernacular name.

Nicobar Island Frog

###### Material examined.

Philippines • 1 individual; Mindanao, Agusan del Sur Province, Municipality of San Francisco, Barangay Caimpugan; 08°24'13"N, 125°52'37"E; MBS 1970 • 9 individuals; Municipality of Rosario, Sitio Sabang–Gibong; 08°19'41"N, 125°53'39"E; BIRC 587–88, 591–93, 817–20 • 10 individuals; Municipality of Bunawan, Barangay San Marcos; confluence of Simulao and Agusan Rivers; 08°14'10"N, 125°55'08"E; BIRC 0610, 825–29, 850–53 • 4 individuals; Municipality of Talacogon, Lake Kasawangan, Barangay La Flora; 08°23'48"N, 125°52'11"E; MBS 2142–43, 2150, 2188 • 2 individuals; Municipality of Loreto, Barangay Poblacion; 08°12'20"N, 125°52'07"E; MBS 2219, 2241.

###### Identification.

A highly pointed snout, a bronze-brown dorsum, bright white markings present on the upper lip, prominent humeral glands positioned on the anteroventral surface of arms, and a creamy venter (Inger 1954; Diesmos et al. 2015; Chandramouli et al. 2020).

###### Remarks.

This introduced species is now very common, apparently ubiquitously distributed, and was observed in dense aggregations actively calling from floating vegetation in freshwater swamps. It also occurs in the peat swamp forest of the Agusan Marsh, where it was observed in smaller choruses of fewer individuals. It is widespread in Southeast Asia, with historical records from the Palawan PAIC (Dumaran Island; Inger 1954) and the Sulu faunal regions (Diesmos et al. 2015), and apparently has been introduced into Agusan Marsh at some point over the last century, from an unknown source; this species is widespread on the land masses of the Sunda Shelf (Malaysia, Indonesia). Recent recognition of the species as a distinct genus *Bijurana* (Chandramouli et al. 2020) has been refuted (Oliver et al. 2015; Chan et al. 2020a), and the newly proposed genus has been placed in synonymy with *Indosylvirana*. Thus, we recognize *I. nicobariensis* (Frost 2025; Chan et al. 2020b).

**Family Rhacophoridae Hoffman, 1932**

##### Kurixalus appendiculatus

Taxon classificationAnimaliaAnuraRhacophoridae

(Günther, 1858)

D5FD6300-037F-5AF0-B2D1-A8354D43A589

Fig. 4L

###### Vernacular name.

Frilled Tree Frog

###### Material examined.

Philippines • 1 individual; Mindanao, Agusan del Sur Province, Municipality of San Francisco, Barangay Caimpugan; 08°24'13"N, 125°52'37"E; KU 314087 • 2 individuals; Municipality of Rosario, Barangay Novele, Sitio Palibo; 08°20'34"N, 125°56'46"E; BIRC 584, 815 • 14 individuals; Municipality of Bunawan, Barangay San Marcos; confluence of Simulao and Agusan Rivers; 08°14'10"N, 125°55'08"E; BIRC 612–15, BIRC 822–24, 843–49 • 1 individual; Municipality of Loreto, Barangay Poblacion; 08°12'20"N, 125°52'07"E; MBS 2231.

###### Identification.

A pale brown, pale gray, or dark gray dorsum, cream to yellow venter, and frilled dermal projections or slight fringes along lateral edges of forearms, tarsi, and feet (Inger 1954; Brown and Alcala 1994; Alcala and Brown 1998; Haas et al. 2018).

###### Remarks.

Numerous individuals of this species occur in areas adjacent to open river systems in freshwater swamps in Agusan Marsh. Populations of this species occur in the eastern island arc of the Philippines (Gonzalez et al. 2013).

##### Philautus acutirostris

Taxon classificationAnimaliaAnuraRhacophoridae

(Peters, 1867)

8DB82AF6-2620-5755-A205-3BAC90F2B961

###### Vernacular name.

Pointed-snout Shrub Frog

###### Material examined.

Philippines • 13 individuals; Mindanao, Agusan del Sur Province, Municipality of San Francisco, Barangay Caimpugan; 08°24'13"N, 125°52'37"E; KU 314065–77.

###### Identification.

A pointed snout in males, and a sharply angular canthal ridge, and possessing gray, brown, or variably patterned, lightly granular dorsum, with dark cruciform markings absent (Brown and Alcala 1994; Alcala and Brown 1998); venter smooth to finely granular.

###### Remarks.

We encountered individuals of this species in the peat swamp forest of Agusan Marsh. The species is also known to occur in montane and disturbed secondary-growth forests in the Mindanao faunal region (Brown and Alcala 1994; Diesmos et al. 2015; Sanguila et al. 2016).

##### Philautus leitensis

Taxon classificationAnimaliaAnuraRhacophoridae

(Boulenger, 1897)

54551304-04B0-5A3D-BE1D-086B8502CDE1

Fig. 4M–P

###### Vernacular name.

Leyte Shrub Frog

###### Material examined.

Philippines • 8 individuals; Mindanao, Agusan del Sur Province, Municipality of San Francisco, Barangay Caimpugan; 08°24'13"N, 125°52'37"E; BIRC 288, MBS 1977–82, 1987 • 2 individuals; Municipality of Rosario, Barangay Novele, Sitio Palibo; 08°20'34"N, 125°56'46"E; BIRC 576, 814 • 1 individual; Municipality of Bunawan, Barangay San Marcos; confluence of Simulao and Agusan Rivers; 08°14'10"N, 125°55'08"E; BIRC 833 • 5 individuals; Municipality of Talacogon, Barangay Zillovia; 08°25'25"N, 125°45'26"E; MBS 1462–66 • 6 individuals; Municipality of Talacogon, Barangay La Flora; 08°23'48"N, 125°52'11"E; MBS 2127–32 • 2 individuals; Municipality of Loreto, Barangay Poblacion; 08°12'20"N, 125°52'07"E; MBS 2217–18.

###### Identification.

Either a yellowish-brown, grayish-green, or dark brown dorsum, with or without a distinct dorsal dark cruciform marking, and few dorsal tubercles, a finely granular venter, and hindlimbs usually with dark transverse crossbars (Inger 1954; Brown and Alcala 1994; Alcala and Brown 1998; Diesmos et al. 2015).

###### Remarks.

This species is morphologically variable (possessing many variable color morphs) and appears to be common in the Katigbok and Caimpugan peat swamp forests of Agusan Marsh.

##### Polypedates leucomystax

Taxon classificationAnimaliaAnuraRhacophoridae

(Gravenhorst, 1829)

00EF7711-137E-5F65-AB3E-F80AB79DC411

Fig. 4Q

###### Vernacular name.

Common Tree Frog

###### Material examined.

Philippines • 1 individual; Mindanao, Agusan del Sur Province, Municipality of San Francisco, Barangay Caimpugan; 08°24'13"N, 125°52'37"E; KU 314078, BIRC 291 • 5 individuals; Municipality of Rosario, Barangay Novele, Sitio Palibo; 08°20'34"N, 125°56'46"E; BIRC 577, 585–86, 816, 821 • 8 individuals; Municipality of Bunawan, Barangay San Marcos; confluence of Simulao and Agusan Rivers; 08°14'10"N, 125°55'08"E; KU 314079, 314080–86 • 8 individuals; Municipality of Bunawan, Barangay San Marcos; confluence of Simulao and Agusan Rivers; 08°14'10"N, 125°55'08"E; BIRC 0603–09, 830 • 1 juvenile; Municipality of Bunawan, Sitio Mambalili; 08°12'24"N, 125°55'30"E; BIRC 0631 • 1 individual; Municipality of Talacogon, Barangay La Flora; 08°23'48"N, 125°52'11"E; MBS 2198 • 1 individual; Municipality of Loreto, Barangay Poblacion; 08°12'20"N, 125°52'07"E; MBS 2282.

###### Identification.

A pale to dark brown dorsum, often with four longitudinal stripes (vague to absent in some individuals), expanded terminal discs of fingers and toes, and a white to cream venter (Brown and Alcala 1994; Alcala and Brown 1998; Diesmos et al. 2015).

###### Remarks.

In Agusan Marsh, individuals of the species occur in the peat swamp forest and in freshwater swamps (actively calling from floating vegetation, grassy banks of pools, and understory branches of small trees and shrubs overhanging these bodies of water).

##### Nyctixalus spinosus

Taxon classificationAnimaliaAnuraRhacophoridae

(Taylor, 1920)

E8422148-36BB-55F1-923B-990E2EB9F74E

Fig. 4R

###### Vernacular name.

Spiny Philippine Tree Frog

###### Material examined.

Philippines • 1 individual; Mindanao, Agusan del Sur Province, Municipality of Talacogon, Barangay Zillovia; 08°25'25"N, 125°45'26"E; MBS 1751.

###### Identification.

An orange- to cinnamon-colored dorsum, covered with numerous, white-tipped asperities and occasional white spots; ventral surfaces bright orange (Taylor 1920a; Brown and Alcala 1994; Alcala and Brown 1998; Diesmos et al. 2015).

###### Remarks.

We encountered a single individual of this species in the Katigbok peat swamp.

### REPTILIA Laurenti, 1768

#### Lizards

**Family Agamidae Spix, 1825**

##### Draco bimaculatus

Taxon classificationAnimaliaAsparagalesAsparagaceae

(Günther, 1864)

F2B68526-9710-5399-81FE-61D89657B75F

###### Vernacular name.

Two-spotted Flying Lizard

###### Material examined.

Philippines • 3 individuals; Mindanao, Agusan del Sur Province, Municipality of San Francisco, Barangay Caimpugan; 08°24'13"N, 125°52'37"E; KU 314088–89, MBS 1971 • 1 individual; Municipality of Talacogon, Lake Kasawangan, Barangay La Flora; 08°23'48"N, 125°52'11"E; MBS 2212.

###### Identification.

A flying lizard with laterally expanded patagia (“wings”) on either side of relatively small body (≤ 70 mm SVL), five free ribs support each patagium; a pair of large black spots on either side of head (with white tubercle at the center), and dorsal coloration of the patagia appearing as a blend of green, yellow, and dark brown (Taylor 1922a; McGuire and Alcala 2000).

###### Remarks.

We encountered individuals of this species in the peat swamp forest of Agusan Marsh. This species is one of the five *Draco* species from Mindanao Island. It occurs sympatrically and syntopically with *D. cyanopterus*, *D. ornatus*, and *D. guentheri*. *Draco bimaculatus* perches closer to the ground than most other sympatric species and occupies disturbed vegetation such as regenerating forest and coconut palm plantations. Thus, it may be sympatric but not syntopic with the forest species *Draco mindanensis* (McGuire and Alcala 2000).

##### Gonocephalus semperi

Taxon classificationAnimaliaPsocodeaPhilopteridae

(Peters, 1867)

766BD89A-BD76-57BB-ABF2-5BC84A71FC51

Fig. 5A

###### Vernacular name.

Southern Philippine Angle-head Forest Dragon

###### Material examined.

Philippines • 1 individual; Mindanao, Agusan del Sur Province, Municipality of Talacogon, Barangay Zillovia; 08°25'25"N, 125°45'26"E; MBS 1457.

**Figure 5.**
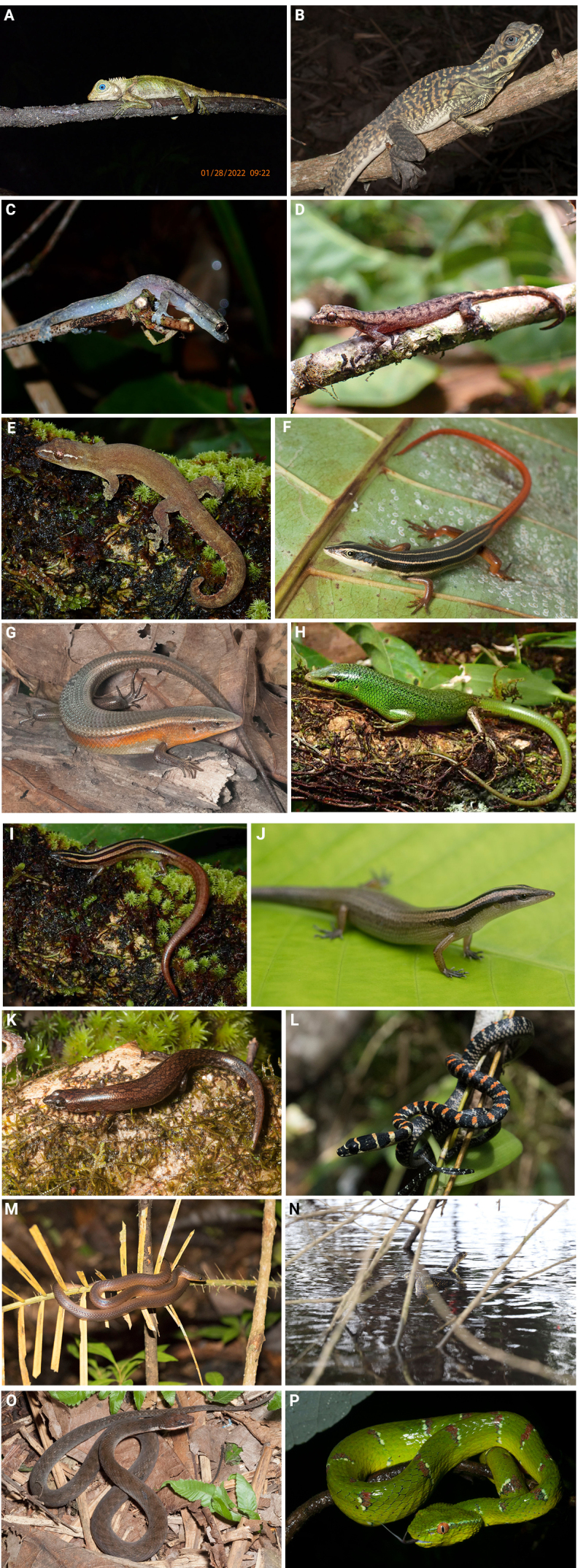
Reptiles recorded from Agusan Marsh Wildlife Sanctuary. **A**. *Gonocephalus interruptus*; **B**. *Hydrosaurus pustulatus*; **C**. *Hemidactylus frenatus*; **D**. *Hemiphyllodactylus typus*; **E**. *Lepidodactylus aureolineatus*; **F**. *Emoia ruficauda*; **G**. *Eutropis multifasciata*; **H**. *Lamprolepis smaragdina*; **I**. *Lipinia quadrivittata*; **J**. *Lipinia semperi*; **K**. *Parvoscincus steerei*; **L**. *Chrysopelea paradisi*; **M**. *Oxyrhabdium modestum*; **N**. *Naja samarensis*; **O**. *Rhabdophis lineatus*; **P**. *Tropidolaemus subannulatus*.

###### Identification.

This is a moderate-sized species, typical appearance to other taxa in the *G. belli* group: it possesses a large head with hypertrophied jaw closure musculature, powerful hindlimbs larger than forelimbs, a large nuchal crest, consisting of enlarged, variably elongated, pointed, and sometimes recurved scales, and which is usually separated by a gap (crest scales absent, or interrupted), from a second, lower vertebral crest (consisting of smaller, less enlarged, dorsal pointed scales), which extends for some length of the trunk. Tail with alternating dark and pale pigmentation, occurring in bands, for the first proximal 50% of its length. Large, mature males typically exhibit bright blue eye coloration (Taylor 1922a; Welton et al. 2017).

###### Remarks.

This species is active during the day but is typically encountered at night while sleeping in a head-up position while grasping trunks of saplings, typically in the vicinity of streams and rivers. In Agusan Marsh, we encountered this species in the Katigbok peat swamp forest.

##### Hydrosaurus pustulatus

Taxon classificationAnimaliaSquamataAgamidae

(Eschscholtz, 1829)

3BB0E5D9-ECEB-5945-9853-EAFC71400DD0

Fig. 5B

###### Vernacular name.

Philippine Sailfin Lizard

###### Material examined.

Philippines • 1 juvenile; Mindanao, Agusan del Sur Province, Municipality of Rosario, Sitio Sabang–Gibong; Agusan River; 08°19'41"N, 125°53'39"E; BIRC 0777.

###### Identification.

A large agamid lizard (SVL 95 mm) with hindlimbs larger than forelimbs, greatly expanded subdigital lamellae underneath the toes, and possessing a unique, prominent, laterally compressed dorsal fin (“sail”) with serrated dorsal edges, extending variably, from above the hips to ≥ 25% proximal length of the tail (Taylor 1922a).

###### Remarks.

A riparian corridor and mangrove forest species (Siler et al. 2014), *H. pustulatus* occurs in overhanging trees, along banks of tributary streams and rivers adjacent to agricultural areas surrounding the freshwater swamp forest of Agusan Marsh.

#### Geckos

**Family Gekkonidae Oppel, 1811**

##### Cyrtodactylus annulatus

Taxon classificationAnimaliaSquamataGekkonidae

(Taylor, 1915)

F9E29FE9-24F8-5DFA-AA23-737888661BAA

###### Vernacular name.

Annulate Bent-toed Gecko

###### Material examined.

Philippines • 5 individuals; Mindanao, Agusan del Sur Province, Municipality of San Francisco, Barangay Caimpugan; 08°24'13"N, 125°52'37"E; KU 314944–46, BIRC 290, MBS 1985 • 8 individuals; Municipality of Talacogon, Barangay Zillovia; 08°25'25"N, 125°45'26"E; MBS 1455, 1458–59, 1756–59, 1777 • 3 individuals; Municipality of Talacogon, Barangay La Flora; 08°23'48"N, 125°52'11"E; MBS 2178, 2205–06.

###### Identification.

A small-sized bent-toed gekkonid lizard, possessing slender digits (i.e., lacking widely expanded subdigital scansors) with recurved claws, a pale gray dorsum crossed by three dark brown to purple barbell-shaped transverse dorsal bands between limb insertions, and possessing precloacal pores only (i.e., lacking a separate series of femoral pores; Brown and Alcala 1978; Welton et al. 2009; Welton et al. 2010a, 2010b).

###### Remarks.

This species is syntopic with *C. agusanensis* in eastern Mindanao (Welton et al. 2010b). In Agusan Marsh, *C. annulatus* occurs in the peat swamp and freshwater swamp forests, where it was encountered on trunks of saplings and understory trees.

##### Gehyra mutilata

Taxon classificationAnimaliaSquamataGekkonidae

(Wiegmann, 1834)

AB478A9C-3FB9-5FCF-B938-9D3FD5FD81DC

###### Vernacular name.

Stump-tailed Gecko

###### Material examined.

Philippines • 1 individual; Mindanao, Agusan del Sur Province, Municipality of Rosario, Barangay Novele, Sitio Palibo; 08°20'34"N, 125°56'46"E; BIRC 871 • 2 individuals; Municipality of Talacogon, Barangay La Flora; 08°23'48"N, 125°52'11"E; MBS 2147–48.

###### Identification.

A small-bodied house gecko, readily identified by its chubby tail (subcylindrical in cross section; ventral surface of tail flattened), which lacks spinose tubercles, homogeneous dorsal body scalation, dorsum pinkish brown, and possesses expanded subdigital scansors underneath the surfaces of fingers and toes configured into a rounded distal adhesive pad when viewed in ventral aspect (Brown and Alcala 1978).

###### Remarks.

This species possesses a distinctive microhabitat preference; it is typically observed in darker areas, on tree trunks or branches, or on man-made structures, but away from lights (unlike other house geckos). In Agusan Marsh, we encountered this species on an abandoned floating house in the freshwater swamp forest.

##### Hemidactylus frenatus

Taxon classificationAnimaliaSquamataGekkonidae

(Duméril & Bibron, 1836)

9D04A1CC-D6EA-56E6-A79C-395513A90720

Fig. 5C

###### Vernacular name.

Common House Gecko

###### Material examined.

Philippines • 2 individuals; Mindanao, Agusan del Sur Province, Municipality of Rosario, Barangay Novele, Sitio Palibo; 08°20'34"N, 125°56'46"E; BIRC 876, 780 • 1 individual; Municipality of Bunawan, Barangay San Marcos; confluence of the Simulao and Agusan Rivers; 08°14'10"N, 125°55'08"E; BIRC 805 • 5 individuals; Municipality of Talacogon, Barangay La Flora; 08°23'48"N, 125°52'11"E; MBS 2163–64, 2175–77.

###### Identification.

A small-bodied house gecko, with a grayish brown to pale brown body, dorsum with small scales and enlarged flat tubercles, and a cylindrical tail (in cross section) with spinose dorsal tail tubercles, and subdigital lamellae underneath fingers and toes forming an elongate, oblong adhesive pad (Taylor 1922a; Brown and Alcala 1978).

###### Remarks.

This species is widespread in the Philippine archipelago and is commonly found in disturbed forested areas close to or inside houses and other artificial structures within human habitations, and typically under electric lights, where they forage for insects. We encountered individuals of this species on an abandoned house and vegetation in the freshwater swamp forest of Agusan Marsh.

##### Hemiphyllodactylus typus

Taxon classificationAnimaliaSquamataGekkonidae

Bleeker, 1860

80A9EE64-5F2B-523F-8C1C-301D8253D70C

Indo-Pacific Slender Gecko Fig. 5D

###### Material examined.

Philippines • 3 individuals; Mindanao, Agusan del Sur Province, Municipality of San Francisco, Barangay Caimpugan; 08°24'13"N, 125°52'37"E; KU 314090–91, MBS 1995 • 1 individual; Municipality of Bunawan, Barangay San Marcos; confluence of Simulao and Agusan Rivers; 08°14'10"N, 125°55'08"E; BIRC 877 • 9 individuals (5 ♀, 4 unsexed) Municipality of Talacogon, Barangay La Flora; 08°23'48"N, 125°52'11"E; MBS 2189–97 • 2 individuals; Municipality of Loreto, Barangay Poblacion; 08°12'20"N, 125°52'07"E; MBS 2221, 2252.

###### Identification.

A very small bodied forest gecko, with a conspicuously elongate, slender body, a prominent lateral stripe from eye to anterior trunk, a dusky tan dorsum (becomes darker when disturbed) with dark blotches in middorsal and postsacral regions, bordered by pale beige transverse bar, creating a distinct coloration transition between the trunk and tail; dorsal scalation homogeneous, and distal subdigital scansors divided (Taylor 1922a; Brown and Alcala 1978; Zug 2010).

###### Remarks.

During our surveys, we found this species to be common in the freshwater swamp forest of Agusan Marsh. Like other gekkonids (e.g., *Hemidactylus*, *Gehyra*), *H. typus* successfully spread via human transport in many islands in the Indo-Pacific Region (Zug 2010; Deso et al. 2020). The Agusan population has been included in a phylogenetic analysis, based on mitochondrial gene sequences, which confirmed its identification as “true” *H. typus* (Grismer et al. 2013).

##### Lepidodactylus aureolineatus

Taxon classificationAnimaliaSquamataGekkonidae

Taylor, 1915

A1429FD5-5EE2-51F6-BB33-D5E32046CD4A

Fig. 5E

###### Vernacular name.

Goldenscaly-toed Gecko

###### Material examined.

Philippines • 6 individuals; Mindanao, Agusan del Sur Province, Municipality of Talacogon, Barangay La Flora; 08°23'48"N, 125°52'11"E; MBS 2117, 2122, 2169–71, 2207 • 4 individuals; Municipality of Loreto, Barangay Poblacion; 08°12'20"N, 125°52'07"E; MBS 2261–64.

###### Identification.

A small-bodied mourning gecko, with a prominent white stripe from snout to forelimb insertion, a yellow dorsum (changes to brown when disturbed), and a tail with denticulate ventrolateral fringes, consisting of slightly enlarged, laterally pointed scales (Taylor 1922a; Brown and Alcala 1978).

###### Remarks.

During our surveys, this species appeared to be common in the freshwater swamp forest of Agusan Marsh, where it was found perched on trunks, branches, or concealed among leaves of dense vines, covering tree trunks, surrounded by swamp water.

#### Skinks

**Family Scincidae Oppel, 1811**

##### Brachymeles orientalis

Taxon classificationAnimaliaSquamataScincidae

Brown & Rabor, 1967

BD260511-99BA-55F6-A926-9ACEE1B25725

###### Vernacular name.

Southern Burrowing Skink

###### Material examined.

Philippines • 5 individuals; Mindanao, Agusan del Sur Province, Municipality of San Francisco, Barangay Caimpugan; 08°24'13"N, 125°52'37"E; KU 314092–96 • 1 individual; Municipality of Talacogon, Barangay Zillovia; 08°25'25"N, 125°45'26"E; MBS 1760.

###### Identification.

A large-bodied, elongate, semi-fossorial scincid lizard, with relatively small, similarly-sized limbs, a brown dorsum, lacking stripes, and a pale orange venter; hands and feet each with five small digits (6 scansors under Finger III, and 10 under Toe IV). Paired supranasals and prefrontals separated (not in medial contact); frontoparietals in medial contact; six supralabials, with 4^th^ or 5^th^ beneath the eye (Siler and Brown 2010).

###### Remarks.

This species occurs in the Caimpugan and Katigbok peat swamp forests of Agusan Marsh. It is one of the pentadactyl Philippine *Brachymeles* (*B. taylori*, *B. schadenbergi*, *B. vindumi*, *B. suluensis*, *B. hilong*, *B. vulcani*, *B. tiboliorum*), and is syntopic with the non-pentadactyl species *B. pathfinderi*, *B. gracilis*, *B. samad*, and *B. boholensis*—all of which occur across the larger and adjacent islands comprising the Mindanao faunal region (Siler et al. 2011).

##### Emoia ruficauda

Taxon classificationAnimaliaSquamataScincidae

Taylor, 1915

CFB81A07-6045-5BAC-9A50-24ED0469AFAD

Fig. 5F

###### Vernacular name.

Redtail Emo Skink

###### Material examined.

Philippines • 9 individuals; Mindanao, Agusan del Sur Province, Municipality of Rosario, Barangay Novele, Sitio Palibo; 08°20'34"N, 125°56'46"E; BIRC 859–60, 863, 866–67, 870, 873–75 • 1 individual; Municipality of Talacogon, Barangay Zillovia; 08°25'25"N, 125°45'26"E; MBS 1778 • 1 individual; Municipality of Talacogon, Barangay La Flora; 08°23'48"N, 125°52'11"E; MBS 2125 • 5 individuals; Municipality of Loreto, Barangay Poblacion; 08°12'20"N, 125°52'07"E; MBS 2243, 2268–71.

###### Identification.

A small-bodied terrestrial skink, hindlimbs substantially larger than forelimbs, possessing a black dorsum, with a golden yellow vertebral stripe from tip of snout to base of tail (which does not merge with the tail color), and a slender, very bright reddish-orange tail (Taylor 1922a; Brown 1991; Gaulke and Alcala 2009).

###### Remarks.

This species occurs in disturbed areas, with thick vegetation, adjacent to freshwater swamps and in the peat swamp forest (see: Gamalinda et al. 2024) of Agusan Marsh. It is one of the two species of *Emoia* (*E. atrocostata*; see Richmond et al. 2021) that occurs in the Philippines, with a disjunct distribution based on occurrence records from the provinces in southern and western portions of the Mindanao faunal region (Brown and Alcala 1980; Gaulke and Alcala 2009; Sy and Buday 2014; Pitogo et al. 2021).

##### Eutropis caraga

Taxon classificationAnimaliaSquamataScincidae

Barley, Diesmos, Siler, Martinez & Brown, 2020

23B7D787-A81A-56B5-B865-A0C81583DD7C

###### Vernacular name.

Caraga Sun Skink

###### Material examined.

Philippines • 4 individuals; Mindanao, Agusan del Sur Province, Municipality of Bunawan, Barangay San Marcos; 08°13'21"N, 125°55'57"E; KU 314105–07, 314098 • 1 juvenile; Municipality of Talacogon, Barangay Zillovia; 08°25'25"N, 125°45'26"E; MBS 1755 • 1 individual; Municipality of Talacogon, Barangay La Flora; 08°23'48"N, 125°52'11"E; MBS 2184.

###### Identification.

A medium-sized terrestrial sun skink of the *E. multicarinata* complex (Brown and Alcala 1980), hindlimbs more robust than forelimbs, with a slender iridescent bronze dorsum, dark lateral coloration, and ≥ 5 keels on each dorsal trunk scale; this species has a combined total of 80–90 subdigital lamellae on the ventral surfaces of fingers and toes (Barley et al. 2020).

###### Remarks.

This species occurs in disturbed areas with sparse vegetation adjacent to freshwater swamps and was also encountered in peat swamp forests of Agusan Marsh. Although some species in the *E. multicarinata* species complex (including *E. caraga*) can be difficult to distinguish from one another without genetic data or reliable occurrence data (known localities), the suite of non-overlapping morphological characteristics evident in our series of *E. caraga* from Agusan Marsh clearly distinguishes this population from species in the *E. indeprensa* complex (including *E. lapulapu*, below; Brown and Alcala 1980). Additionally, some of our specimens have been genetically identified (as an independent validation of the identifications we made based on morphological characters) and were designated by Barley et al. (2020) as paratypes in the descriptions of *E. caraga*. Lastly, populations from Dinagat and Siargao (small separate islands of eastern Mindanao) are demonstrably genetically distinct (Barley et al. 2020) and should be the focus of future taxonomic scrutiny.

##### Eutropis lapulapu

Taxon classificationAnimaliaSquamataScincidae

Barley, Diesmos, Siler, Martinez & Brown, 2020

BDD32899-57C5-56CB-B4E6-0EB4993748F3

###### Vernacular name.

Lapu–lapu’s Sun Skink

###### Material examined.

Philippines • 1 individual; Mindanao, Agusan del Sur Province, Municipality of San Francisco, Barangay Caimpugan; 08°24'13"N, 125°52'37"E; KU 314104.

###### Identification.

A small- to medium-sized terrestrial sun skink of the *E. indeprensa* complex (Brown and Alcala 1980), with hindlimbs more robust than forelimbs, and a bronze to olive dorsum, ≤ 9keels per dorsal body scales, and with a combined total of 70–80 subdigital lamellae on ventral surfaces of fingers and toes (Barley et al. 2020).

###### Remarks.

This species occurs in the peat swamp forests of Agusan Marsh. It is widespread in the Mindanao faunal region, but it also occurs on southern Luzon, Cebu, and Panay (Barley et al. 2020). Although some species in the *E. indeprensa* species complex (including *E. lapulapu*) can be difficult to distinguish from one another without genetic data or reliable occurrence data (known localities), the suite of non-overlapping morphological characteristics evident in our series of *E. lapulapu* from Agusan Marsh clearly distinguishes this population from species in the *E. multicarinata* complex (including *E. caraga*, above; Brown and Alcala 1980). Additionally, a portion of our specimens from Agusan Marsh have had their identifications genetically confirmed (as an independent check, or validation of our identification, which were based on morphology), and were designated by Barley et al. (2020) as paratypes in the descriptions of *E. lapulapu*.

##### Eutropis multifasciata

Taxon classificationAnimaliaSquamataScincidae

(Kuhl, 1820)

C40154BD-62BC-58DF-8671-562FDDF9D508

Fig. 5G

###### Vernacular name.

Common Sun Skink

###### Material examined.

Philippines • 1 individual; Mindanao, Agusan del Sur Province, Municipality of San Francisco, Barangay Caimpugan; 08°24'13"N, 125°52'37"E; KU 314099 • 2 individuals; Municipality of Bunawan, Barangay San Marcos; 08°13'21"N, 125°55'57"E; KU 314100, 314103; 2 individuals; same locality as for preceding; 08°24'13"N, 125°55'57"E; KU 314101–02 • 1 individual; Municipality of Rosario, Barangay Novele, Sitio Palibo; 08°20'34"N, 125°56'46"E; BIRC 872 • 2 individuals; Municipality of Talacogon, Barangay Zillovia; 08°25'25"N, 125°45'26"E; MBS 1456, 1460 • 5 individuals; Municipality of Talacogon, Barangay La Flora; 08°23'48"N, 125°52'11"E; MBS 2121, 2126, 2146, 2165, 2184 • 3 individuals; Municipality of Loreto, Barangay Poblacion; 08°12'20"N, 125°52'07"E; MBS 2236, 2254–55.

###### Identification.

A very large-bodied terrestrial sun skink, hindlimbs larger than forelimbs, with a robust, thickened body, and proportionally long tail. Dorsum olive brown to gray, with dark dorsolateral stripes; venter cream to pale gray; most dorsal and lateral body scales with three weak keels (Barley et al. 2020).

###### Remarks.

This species is common in disturbed areas adjacent to, and in freshwater swamps, and individuals were also encountered in the peat swamp forest of Agusan Marsh. It is widespread throughout the various islands of the Philippine archipelago (Brown and Alcala 1980; Barley et al. 2014, 2015).

##### Lamprolepis smaragdina

Taxon classificationAnimaliaLepidopteraLimacodidae

(Lesson, 1829)

E55CA15E-2432-5248-86A8-9BFB1B594043

Fig. 5H

###### Vernacular name.

Green Tree Skink

###### Material examined.

Philippines • 2 individuals; Mindanao, Agusan del Sur Province, Municipality of Bunawan, Barangay San Marcos; 08°13'21"N, 125°55'57"E; KU 314108–09 • 1 individual; Municipality of Rosario, Barangay Novele, Sitio Palibo; 08°20'34"N, 125°56'46"E; BIRC 768 • 1 ♀; Municipality of Bunawan, Barangay San Marcos; confluence of Simulao and Agusan Rivers; 08°14'10"N, 125°55'08"E; BIRC 626 • 1 individual; Municipality of Talacogon, Barangay Zillovia; 08°25'25"N, 125°45'26"E; MBS 1454 • 7 individuals; Municipality of Talacogon, Barangay La Flora; 08°23'48"N, 125°52'11"E; MBS 2116, 2124, 2179–83 • 2 individuals; Municipality of Loreto, Barangay Poblacion; 08°12'20"N, 125°52'07"E; MBS 2256–57.

###### Identification.

A large-bodied arboreal skink, with a slender, elongate body, long tail, and acutely pointed snout, and a uniquely enlarged, bright orange heel scale present in males; this species has an emerald green anterior half of the body, fading to gray or brown posteriorly, on dorsal surfaces of trunk and hindlimbs; venter pale greenish cream (Taylor 1922a; Brown and Alcala 1980).

###### Remarks.

This species inhabits disturbed areas adjacent to freshwater swamp and peat swamp forests of Agusan Marsh. Taylor (1922a) previously recognized this species as *Dasia smaragdinum* (in addition to two other subspecies: *D. olivaceum olivaceum* and *D. olivaceum semicincta*), but it has since been revised to *L. smaragdina* (Brown and Alcala 1980), which is known to consist of two deeply divergent clades (not each other’s closest relatives), most likely representing two separate invasions of the archipelago. Our specimen likely belongs to the clade documented from Mindanao, Palawan, Camiguin Sur, Siquijor, and the Indonesian island of Salibabu (Linkem et al. 2013); the other monophyletic and phylogenetically distinct clade is widely distributed throughout most of the remaining islands spanning the archipelago (Linkem et al. 2013).

##### Lipinia pulchella

Taxon classificationAnimaliaSquamataScincidae

(Gray, 1845)

F544B0F8-743A-5503-8E29-45E8735C2CB5

###### Vernacular name.

Beautiful Lipinia

###### Material examined.

Philippines • 3 individuals; Mindanao, Agusan del Sur Province, Municipality of Bunawan, Barangay San Marcos; 08°13'21"N, 125°55'57"E; KU 314121–23 • 14 individuals; Municipality of San Francisco, Barangay Caimpugan; 08°24'13"N, 125°52'37"E; KU 314110–20, MBS 1972–73, 1984 • 3 individuals; Municipality of Rosario, Barangay Novele, Sitio Palibo; 08°20'34"N, 125°56'46"E; BIRC 861, 868–69 • 1 individual; Municipality of Loreto, Barangay Poblacion; 08°12'20"N, 125°52'07"E; MBS 2224.

###### Identification.

A small-bodied arboreal tree skink, with an elongate, slender body, thin limbs (hindlimbs larger), acutely pointed snout, pale brown dorsum with a yellow middorsal stripe, and with long slender digits (24–31 subdigital lamellae under toe IV) and an exposed tympanum (external auricular opening visible) (Taylor 1922a; Brown and Alcala 1980; Das and Austin 2007).

###### Remarks.

This species occurs in the peat swamp forest of Agusan Marsh, where it can be found among branches or hiding among leaves of vines covering small trees, surrounded by water.

##### Lipinia quadrivittata

Taxon classificationAnimaliaSquamataScincidae

(Peters, 1867)

88E3D6BF-1DBD-5D1C-8759-D2504FD7D837

Fig. 5I

###### Vernacular name.

Four-striped Lipinia

###### Material examined.

Philippines • 2 individuals; Mindanao, Agusan del Sur Province, Municipality of San Francisco, Barangay Caimpugan; 08°24'13"N, 125°52'37"E; KU 314124–25 • 8 individuals; Municipality of Talacogon, Barangay La Flora; 08°23'48"N, 125°52'11"E; MBS 2119–20, 2123, 2161, 2201–04 • 9 individuals; Municipality of Loreto, Barangay Poblacion; 08°12'20"N, 125°52'07"E; MBS 2213–14, 2223, 2225–26, 2244–47.

###### Identification.

A small-bodied arboreal tree skink, with an elongate, slender body, thin limbs (hindlimbs larger), moderately pointed snout, and a brown dorsum marked with four distinct dark longitudinal stripes (two each on each side of the spine, from eye to base of tail), a hidden tympanum (external auricular opening absent), and 15 or 16 lamellae under toe IV (Taylor 1922a; Brown and Alcala 1980).

###### Remarks.

This species is found on the bark of trees in the peat swamp forest of Agusan Marsh and was frequently observed and/ or captured among the ferns of trees inundated by swamp water.

##### Lipinia semperi

Taxon classificationAnimaliaSquamataScincidae

(Peters, 1867)

41CD0E73-2832-5380-9507-289FB7F4730D

Fig. 5J

###### Vernacular name.

Semper’s Lipinia

###### Material examined.

Philippines • 6 individuals; Mindanao, Agusan del Sur Province; Municipality of Loreto, Barangay Poblacion; 08°12'20"N, 125°52'07"E; MBS 2248–50, 2272–74.

###### Identification.

A small arboreal tree skink (relatively large for a species of *Lipinia*), with a more robust, broader trunk and thicker limbs (hindlimbs larger), dorsum brown-olive to dark brown with four dorsolateral black stripes (2 on each side of the spine), extending from tip of snout, or the eyes, narrowing posteriorly, to a point ~ ¾ the length of the axilla-groin region, then fading to become indistinguishable from background pigmentation. Prefrontals not in contact, tympanum exposed, four supraoculars, and seven supralabials (5^th^ beneath the center of eye), 24 pad-like lamellae beneath toe IV, and a pale brown venter (Taylor 1922a; Brown and Alcala 1980).

###### Remarks.

This species appears to be common in the freshwater swamp forest of the Agusan Marsh, where it was frequently observed and/or captured among branches or while hiding on vines covering small understory saplings inundated by swamp water.

##### Parvoscincus steerei

Taxon classificationAnimaliaSquamataScincidae

(Stejneger, 1908)

4D8A8186-C222-5BAA-8917-21EDFC0592D1

Fig. 5K

###### Material examined.

Philippines • 2 individuals; Mindanao, Agusan del Sur Province, Municipality of San Francisco, Barangay Caimpugan; 08°24'13"N, 125°52'37"E; KU 314126–27.

###### Identification.

A very small–sized forest skink (26.4 to 36.0 mm for mature males; 27.5 to 35.5 for mature females; Brown and Alcala 1980), with a blunt, short snout, and a relatively large eye. Coloration is homogeneous dark brown, with paler dorsolateral stripes or a series of pale blotches. Ventral coloration pale cream; tail coloration indistinguishable from overall dark brown body color. This species has four supraoculars, seven upper labials, 28–32 midbody scale rows, and 10–13 subdigital lamellae beneath toe IV (Brown and Alcala 1980).

###### Remarks.

This secretive and inconspicuous forest floor species is encountered underneath leaf litter or forest floor debris, beneath logs, and occasionally underneath rocks in the vicinity of streams; it has been recorded from both primary and original, regenerating forest (Brown and Alcala 1980), consistent with our own observations within the peat swamp forest in Agusan Marsh.

##### Pinoyscincus jagori

Taxon classificationAnimaliaSquamataScincidae

(Peters, 1864)

E2382EDD-281A-5EA6-8587-68CFA6B30560

###### Material examined.

Philippines • 1 individual; Mindanao, Agusan del Sur Province, Municipality of Rosario, Barangay Novele, Sitio Palibo; 08°20'34"N, 125°56'46"E; BIRC 880.

###### Identification.

A medium-sized terrestrial forest skink, with hindlimbs substantially more robust than forelimbs, dorsum medium brown with irregular black markings and, in some specimens, a broken (disrupted) black vertebral stripe; venter ivory to cream colored. Body scales unkeeled and relatively uniform throughout; prefrontals separate, frontoparietals and parietal pairs both in medial contact. This species has four supraoculars (1^st^ and 2^nd^ in contact with frontal, 3^rd^ and 4^th^ with frontoparietal), nuchals undifferentiated, paravertebral scales in 56–80 rows, midbody scales in 30–44 rows, and 17–26 subdigital lamellae beneath toe IV (Taylor 1922a; Linkem et al. 2010).

###### Remarks.

In their definition of *P. jagori*, Brown and Alcala (1980) emphasized (and illustrated) two vertical black bars crossing the last supralabial scales, beneath the eyes, as the key characteristic for identifying the species. However, with much larger sample sizes (and a molecular phylogeny to confirm identifications), Linkem et al. (2010) demonstrated a gradual, continuous range of color pattern variation, from some populations with two bold bars beneath the eyes to others with immaculate white upper labial scales (dark bars absent), and some exhibiting intermediate states in these color patterns. Nevertheless, using Brown and Alcala’s (1980) key and relying on their reported scalation characters, we confidently arrive at the identification of our specimens as *P. jagori*. Mindanao specimens of this species included in the Linkem et al. (2010) phylogenetic analysis had strong support in their “jagori Clade 4"—which we emphasize here, to maximize future reproducibility of our results, given that specimens (from islands other than Mindanao) identified by Linkem et al. (2010) using the Brown and Alcala (1980) key came out in three highly divergent clades, which Linkem et al. (2010) interpreted as the probable existence of unrecognized diversity (likely morphologically cryptic new species).

##### Sphenomorphus fasciatus

Taxon classificationAnimaliaSquamataScincidae

(Gray, 1845)

02C7390B-74D1-58C8-AED5-A0AB12428AA6

###### Vernacular name.

Banded Forest Skink

###### Material examined.

Philippines • 1 individual; Mindanao, Agusan del Sur Province, Municipality of Loreto, Barangay Poblacion; 08°12'20"N, 125°52'07"E; MBS 2253.

###### Identification.

A medium-sized terrestrial forest skink, with proportionally elongate trunk, unkeeled dorsal scales, a brownish-black dorsum crossed by thin, yellow, white, or pale blue transverse bands; venter immaculate ivory. This species is also uniquely identified by its proximally thickened tail base and gradually tapering distal portions of its tail (Taylor 1922a; Brown and Alcala 1980).

###### Remarks.

This Mindanao PAIC endemic species appears to have evolved because of dispersal and colonization of the southern Philippines from New Guinea by way of eastern Indonesia (Linkem et al. 2011).

#### Monitor lizards

**Family Varanidae Gray, 1827**

##### Varanus cumingi

Taxon classificationAnimaliaSquamataVaranidae

Martin, 1839

EC84F783-E76B-53AB-AFAC-89E19926BB68

###### Vernacular name.

Cuming’s Water Monitor Lizard

###### Material examined.

Philippines • 1 individual; Mindanao, Agusan del Sur Province, Municipality of Bunawan, Barangay San Marcos; 08°13'21"N, 125°55'57"E; KU 314128.

###### Identification.

This large-bodied, robust monitor lizard possesses the typical attributes of monitor lizards: a robust body, extremely developed, powerful limbs, strongly recurved claws, a long, muscular neck, and an enlarged head with big eyes and an alert disposition. *Varanus cumingi* is average-sized for Philippine members of the *V. salvator* group, but is strikingly colored/patterned, possessing uniquely bright yellow coloration, especially on the head, and entirely unlike species to the north (Gaulke 1991, 1992; Welton et al. 2014).

###### Remarks.

Philippine species of the *Varanus salvator* group (Gaulke 1991, 1992) have been the subject of continued taxonomic study, resulting in the recognition of five Philippine endemic species (Koch et al. 2007, 2010; Welton et al. 2013, 2014). Welton et al. (2014) elevated the former subspecies *Varanus cumingi samarensis* (Koch et al. 2007, 2010) to the full-species level, based on multilocus genetic data and statistical species delimitation analyses. Welton et al. (2014) also diagnosed populations from Samar and Leyte islands as *Varanus samarensis* (based on discrete, discontinuous, color patterns character state differences), a distinct species that is readily distinguished from *Varanus cumingi*, the Mindanao-endemic species, which we encountered in Agusan Marsh.

#### Snakes

**Family Colubridae Oppel, 1811**

##### Boiga dendrophila latifasciata

Taxon classificationAnimaliaSquamataColubridae

(Boulenger, 1896)

222056F1-4B82-528F-A6B3-E1EA272FC1E1

###### Vernacular name.

Gold-ringed Cat-eyed Mangrove Snake

###### Material examined.

Philippines • 2 individuals; Mindanao, Agusan del Sur Province, Municipality of Talacogon, Barangay La Flora; 08°23'48"N, 125°52'11"E; MBS 2209–10.

###### Identification.

A medium- to large-sized cat-eyed mangrove snake (SVL 561–964 mm) with a vertical pupil and a black body interspersed with yellow crossbands (each ≥ 2 scale rows wide) from neck to tail, becoming wider on the lateral surfaces of the body (Weinell et al. 2019).

###### Remarks.

This species was encountered at night, perched on the upper branches (crown) of a 3-m tree in a freshwater swamp. The three other Philippine subspecies of *Boiga dendrophila* include *B. d. divergens* (Luzon PAIC islands), *B. d. multicincta* (Palawan PAIC), and *B. d. levitoni* (West Visayan PAIC), in addition to four non-Philippine subspecies (Weinell et al. 2019; Uetz et al. 2025)—not all of which are consistently supported as monophyletic by Weinell et al.’s (2020) recent multilocus phylogenetic analysis of the genus.

##### Chrysopelea paradisi

Taxon classificationAnimaliaSquamataColubridae

H. Boie in F. Boie, 1827

EC1C6EA2-D010-589F-B4F3-A3EFC02A1C1E

Fig. 5L

###### Vernacular name.

Paradise Tree Snake

###### Material examined.

Philippines • 1 juvenile; Mindanao, Agusan del Sur Province, Municipality of Talacogon, Barangay La Flora; 08°23'48"N, 125°52'11"E; photographic voucher (juvenile) at FSUU (ID in published dataset: FSUU-BIRC-AMWS-TS-337).

###### Identification.

A “flying” snake with a circular pupil, 17 scale rows sat midbody (the outer one [most ventral] with a prominent notch at the hinged margin with ventral) and a black dorsum interspersed with reddish-yellow transverse bars (Weinell et al. 2019).

###### Remarks.

We encountered this species in the freshwater swamp forest of Agusan Marsh, where it was active during the day.

##### Coelognathus erythrurus erythrurus

Taxon classificationAnimaliaSquamataColubridae

(Duméril, Bibron & Duméril, 1854)

BCAA3BBD-B40D-5038-B286-C2664EFF1C41

###### Vernacular name.

Philippine Rat Snake

###### Material examined.

Philippines • 1 individual; Mindanao, Agusan del Sur Province, Municipality of Talacogon, Barangay La Flora; 08°23'48"N, 125°52'11"E; MBS 2211.

###### Identification.

A large-sized Rat Snake (SVL 1.131 m) with a circular pupil, dorsal and ventral body color a combination of generally brownish-olive body, yellowish in the nuchal region (visible dorsolaterally on labials), and reddish-brown from midbody to tail; dorsal midbody scales in 21 longitudinal rows, 219 ventral body scales, and 65 subcaudal scales (Weinell et al. 2019).

###### Remarks.

This species was encountered in the freshwater swamp forest of Agusan Marsh. Apart from two other Philippine subspecies (*C. e. manillensis* [Luzon and Mindoro PAICs, Babuyan and Batanes islands] and *C. e. psephenourus* [West Visayan PAIC]), the only non-Philippine populations of this species have been recorded on the Indonesian island of Sulawesi (*C. e. celebensis*; Leviton 1979; Leviton et al. 2018).

##### Dendrelaphis marenae

Taxon classificationAnimaliaSquamataColubridae

Vogel & Van Rooijen, 2008

3A496CC9-1A34-590D-A741-319C03D08C07

###### Vernacular name.

Maren’s Bronze-back Tree Snake

###### Material examined.

Philippines • 1 individual; Mindanao, Agusan del Sur Province, Municipality of San Francisco, Barangay Caimpugan; 08°24'13"N, 125°52'37"E; KU 314130 • 1 individual; Municipality of Rosario, Barangay Novele, Sitio Palibo; 08°20'34"N, 125°56'46"E; BIRC 862 • 1 ♀; Municipality of Bunawan, Barangay San Marcos; confluence of Simulao and Agusan Rivers; 08°14'10"N, 125°55'08"E; BIRC 595 • 1 individual; Municipality of Talacogon, Barangay Zillovia; 08°25'25"N, 125°45'26"E; MBS 1766.

###### Identification.

A species of *Dendrelaphis* (SVL 471–683 mm) with a bronze- and black-greenish dorsum, 15 longitudinal rows of dorsal scales at midbody, and vertebral scales that are enlarged relative to other dorsal body scales (Weinell et al. 2019).

###### Remarks.

This widespread Philippine species occurs throughout the Mindanao PAIC islands of Mindanao, Samar, Leyte, and Bohol (and smaller islands associated with these landmasses (Leviton et al. 2018) and was observed at the edges of peat swamp and freshwater swamp forests of Agusan Marsh.

##### Dendrelaphis philippinensis

Taxon classificationAnimaliaSquamataColubridae

(Günther, 1879)

3A0F4656-68D4-521E-B7CB-1BE1BC02E681

###### Vernacular name.

Philippine Bronze-back Tree Snake

###### Material examined.

Philippines • 2 ♂, 1 ♀; Mindanao, Agusan del Sur Province, Municipality of Bunawan, Barangay San Marcos; confluence of Simulao and Agusan Rivers; 08°14'10"N, 125°55'08"E; BIRC 594, 596, 878.

###### Identification.

A species of *Dendrelaphis* (SVL 601–660 mm) with 13 longitudinal rows of dorsal scales at midbody, a pale ventrolateral body stripe, and two black longitudinal stripes at midbody: the 1^st^ between ventral scales and first row of dorsal body scales, and the 2^nd^ on anterior one-fifth of the body along the border of second and third dorsal body scale rows (Leviton 1968; Weinell et al. 2019).

###### Remarks.

This widespread southeastern island arc (Mindanao faunal region; it is replaced north of Samar Island by *D. luzonensis*, the Luzon faunal region endemic) Philippine species (Leviton et al. 2018) occurs in disturbed areas adjacent to peat swamp and freshwater swamp forests of Agusan Marsh.

**Family Cyclocoridae Weinell & Brown, 2017**

##### Oxyrhabdium modestum

Taxon classificationAnimaliaSquamataCyclocoridae

(Duméril, 1853)

39C1F7C3-F54B-5002-9157-F6C27241E565

Fig. 5M

###### Vernacular name.

Philippine Shrub Snake

###### Material examined.

Philippines • 1 individual; Mindanao, Agusan del Sur Province, Municipality of Rosario, Barangay Novele, Sitio Palibo; 08°20'34"N, 125°56'46"E; BIRC 763 • 1 individual; Municipality of Bunawan, Barangay San Marcos; confluence of Simulao and Agusan Rivers; 08°14'10"N, 125°55'08"E; BIRC 879 • 4 individuals; Municipality of Talacogon, Barangay Zillovia; 08°25'25"N, 125°45'26"E; MBS 1761–64.

###### Identification.

A medium-sized snake with a distinctly pointed (narrow, elongate) snout, protruding round eyes, anterior chin shields much larger than posterior chin shields, loreal scale separate from the second supralabial, eight supralabial scales (5^th^ and 6^th^ bordering eye), 15 longitudinal dorsal scale rows at midbody and dorsum with smooth, reddish-brown scales throughout (Leviton 1964; Weinell et al. 2019).

###### Remarks.

This species is widespread on Mindanao and southern Luzon PAIC landmasses (Leviton et al. 2018) and occurs in disturbed areas adjacent to freshwater swamp, or on the forest floor of peat swamp forests in Agusan Marsh.

**Family Elapidae Boie, 1827**

##### Naja samarensis

Taxon classificationAnimaliaSquamataElapidae

Peters, 1861

5187C1D2-3371-54F1-B34F-772C5BE8A14B

Fig. 5N

###### Vernacular name.

Samar Cobra

###### Material examined.

Philippines • 2 individuals; Mindanao, Agusan del Sur Province, Municipality of Talacogon, Barangay La Flora; 08°23'48"N, 125°52'11"E; MBS 2144–45.

###### Identification.

A large-sized snake with a distinctly expandable flattened “hood” when threatened in life, and with a black dorsum interspersed with yellow anterior ventral body scales followed by black bands that gradually fade posteriorly, three postocular scales, a postnasal scale that is separate from the prefrontal scale, 17–19 longitudinal rows of dorsal scales at midbody, and 162–168 ventral body scales (Leviton 1964; Weinell et al. 2019).

###### Remarks.

This species is widespread among Mindanao PAIC landmasses and was encountered and captured in forest and agricultural areas—but was also observed twice swimming in the water of seasonally inundated freshwater swamp forests of Agusan Marsh.

**Family Natricidae Bonaparte, 1838**

##### Rhabdophis lineatus

Taxon classificationAnimaliaSquamataColubridae

(Peters, 1861)

E111BCB2-B11C-5A74-BF9A-354DFE0BB210

Fig. 5O

###### Vernacular name.

Zigzag-lined Water Snake

###### Material examined.

Philippines • 1 individual; Mindanao, Agusan del Sur Province, Municipality of Rosario, Barangay Novele, Sitio Palibo; 08°20'34"N, 125°56'46"E; BIRC 767.

###### Identification.

A medium-sized snake (SVL 390–440 mm) with a dark gray, reddish-brown, or brown dorsum, 17 longitudinal rows of strongly keeled dorsal scales at midbody, two preocular scales, a distinct white stripe across supralabials, one anterior temporal scale in contact with the sixth supralabial scale, and venter pale cream to white (Weinell et al. 2019).

###### Remarks.

This species is widely distributed across the Mindanao PAIC faunal region (Leviton et al. 2018) and was observed at night, actively hunting in a dry stream bed.

**Family Psammodynastidae Das, Greenbaum, Brecko, Pauwels, Ruane, Pirro & Merilä, 2024**

##### Psammodynastes pulverulentus

Taxon classificationAnimaliaSquamataPseudaspididae

(Boie, 1827)

27BA5A75-6712-5FAA-A0E8-84A9AD87A718

###### Vernacular name.

Common Mock Viper

###### Material examined.

Philippines • 1 individual; Mindanao, Agusan del Sur Province, Municipality of San Francisco, Barangay Caimpugan; 08°24'13"N, 125°52'37"E; KU 314132.

###### Identification.

A medium-sized snake with variably brown with scattered, black, gray, cream, and white markings, loreal and preocular scales present, pupil round, supraoculars greatly enlarged (resulting in a general, pit viper-like appearance), nasal scales undivided, dorsal body scales smooth, in 17 longitudinal rows at midbody, and subcaudal scales paired (Weinell et al. 2019).

###### Remarks.

This species is widespread on all major Philippine islands (Leviton et al. 2018) and was observed in the peat swamp forests of Agusan Marsh.

**Family Pareidae Romer, 1956**

##### Aplopeltura boa

Taxon classificationAnimaliaSquamataPareidae

(Boie, 1828)

80D9725F-CFEB-5876-B2C0-DB8A4CFCA1B9

###### Vernacular name.

Blunt-headed Slug-eating Snake

###### Material examined.

Philippines • 1 individual; Mindanao, Agusan del Sur Province, Municipality of Bunawan, Barangay San Marcos; 08°13'21"N, 125°55'57"E; KU 314129.

###### Identification.

A long, slender-bodied arboreal snake with smooth dorsal scales in 13 rows at midbody, large eyes with round pupils, a laterally compressed head, unusually squarish, blunt snout, ventral body scales larger than dorsal body scales, tail conical or rounded (Weinell et al. 2019), and dorsal body surfaces irregularly patterned in patches of brown, tan, and black.

###### Remarks.

This species is widely distributed across Mindanao PAIC islands (Leviton et al. 2018) and was encountered climbing in shrub layer vegetation on the forest edge, following heavy rain.

**Family Viperidae Oppel, 1811**

##### Tropidolaemus subannulatus

Taxon classificationAnimaliaSquamataViperidae

(Gray, 1842)

E06C52A7-F757-5B63-B2B3-E81E0508D3C7

Fig. 5P

###### Vernacular name.

Eastern Temple Pit viper

###### Material examined.

Philippines • 1 individual; Mindanao, Agusan del Sur Province, Municipality of San Francisco, Barangay Caimpugan; 08°24'13"N, 125°52'37"E; BIRC 289 • 1 individual; Municipality of Talacogon, Barangay Zillovia; 08°25'25"N, 125°45'26"E; MBS 1765.

###### Identification.

A small but robust, heavy-bodied pit viper (SVL 250–339 mm), with flat, broadly triangular head, scales on lateral and ventral head surfaces strongly keeled, a grayish blue postocular stripe, and 9–16 head scales between supraoculars, dorsum pale to yellowish-green, interspersed with red and white crossbands, 22 longitudinal midbody scale rows, second supralabial scale not in contact with scale forming anterior border of heat sensing pit, and third supralabial separated from subocular by two scales (Weinell et al. 2019).

###### Remarks.

This species is widely distributed throughout the southeastern Philippines (Leviton et al. 2018) and was encountered perched in understory trees of peat swamp forests of Agusan Marsh.

#### Turtles

**Family Geoemydidae Theobald, 1868**

##### Cuora philippinensis

Taxon classificationAnimaliaTestudinesGeoemydidae

Blanck, Gaillard, Protiva, Wheatley, Shi, Liu, Ray & Anders, 2023

C3774D0F-FD85-5242-BFB3-31C8B1373108

###### Vernacular name.

Philippine Box Turtle

###### Material examined.

Philippines • 1 individual; Mindanao, Agusan del Sur Province, Municipality of Rosario, Barangay Novele, Sitio Palibo; 08°20'34"N, 125°56'46"E; BIRC 774 • 1 individual; Municipality of Talacogon, Barangay La Flora; 08°23'48"N, 125°52'11"E; MBS 2208 (salvaged).

###### Identification.

A freshwater box turtle (plastron length 95–139 mm), carapace with vertebral keels and slightly serrated posterior margin, plastron has prominent black nearly completely closing shell in adult, head uniform dark brown above continuing on the neck, a dorsolateral yellow stripe from point of snout, along canthus rostralis, and through the upper part of the orbit, bordered below by a dark brown line which runs through the eye (Taylor 1920a, b; Diesmos et al. 2008).

###### Remarks.

This species is widely distributed throughout the Philippines and occurs locally in disturbed and cultivated areas adjacent to the freshwater swamp forest of Agusan Marsh. Individuals are often kept as pets by the local people. Recently distinguished from the widespread Southeast Asian species *C. amboinensis*, the Philippine freshwater box turtle *C. philippinensis* appears most closely related to populations from Sulawesi and the Moluccas, Indonesia (Blanck et al. 2023).

## Discussion

This study contributes to the amelioration of the basic scientific knowledge shortcomings of the amphibian and reptile fauna of the mixed swamp and peat swamp forests of the Agusan Marsh Wildlife Sanctuary (AMWS), of eastern Mindanao Island, southern Philippines. For this purpose, we relied on a century-long survey-resurvey effort and updated the cumulative attempt towards arriving at a full, comprehensive inventory of this fauna. The biodiversity of swamp and peat swamp forests of Southeast Asia is continually threatened by habitat modification and exploitative resource extraction activities of humans, and yet remains immensely understudied (Adila et al. 2017; Giesen et al. 2018; Harrison and Rieley 2018).

Tropical swamp and peat swamp forests constitute habitat types with emergent vegetation, well-adapted to flood plains (Giesen et al. 2018), with vertebrate faunas composed of habitat specialists, disturbance-tolerant species, and numerous threatened species (Posa et al. 2011; Page and Rieley 2016). We did not note any amphibian or reptile species that are entirely peat swamp-dependent species (see Posa et al. 2011). However, we confirm the presence of swamp habitat-dependent species, like the native amphibians *Limnonectes leytensis*, *Kurixalus appendiculatus*, *Fejervarya vittigera*, and the introduced species *Indosylvirana nicobariensis* (Sanguila et al. 2016; AmphibiaWeb 2025; Frost 2025), and native aquatic habitat specialist reptiles like *Hydrosaurus pustulatus*, *Boiga dendrophila*, and *Cuora philippinensis*, which utilize swamps (Sanguila et al. 2016; Uetz et al. 2025).

Comparison of the number of species among sites (for surveys summarized here) would benefit from more standardized survey periods and species observed (Gotelli and Colwell 2001; Magurran 2004). The species accumulation curves (Fig. 3B) indicate that sampling completeness is not 100% in the AMWS and that, in all habitat types, more species will likely be observed with additional surveys. We observed a similar species richness in different habitat types, but the mean Shannon Diversity Index was lower for peat swamps, likely due to a more uneven distribution of sample species in peat swamps. Notably, we find that the total diversity observed in only 15 days in 2008 was greater than what was observed in 35 days across three sampling years (2019, 2021, 2022). Although this may indicate a decline in diversity due to habitat degradation, more consistent survey efforts over longer periods of time are required to quantify species richness and diversity indices more accurately (Magurran 2004).

We report an important additional new Philippine distributional record for *Indosylvirana nicobariensis*, extending its range of occurrence within the Mindanao faunal region for the first time. This species is widespread in Sundaland and the Nicobar Islands (Oliver et al. 2015; Chandramouli et al. 2020), but in the Philippine archipelago, until this report, it was known to occur only in Palawan and, possibly, Sulu PAIC faunal regions (Diesmos et al. 2015; Frost 2025). This species’ new occurrence record in Agusan Marsh extends its known Philippine range more than 700 km from the previous documented point of occurrence, namely Dumaran Island, Palawan, and nearly 600 km from older records in the Sulu Archipelago (Taylor 1920a; Taylor 1922a; Inger 1954; Diesmos et al. 2015; Frost 2025). The species appears to prefer swamp habitats and is tolerant of frequent habitat disturbance, such as periodic agricultural habitat modification (Chandramouli et al. 2020), which may explain the ability of *I. nicobariensis* to colonize and persist, following what we presume has been a human-facilitated introduction (the species was notably absent, but would have been recorded by Taylor if it had been present in the early 1900s) in disturbed mixed swamp forests, close to open water ecosystem habitats (Agusan and Simulao rivers).

We also report the rediscovery of the red-tailed swamp skink (*Emoia ruficauda*) in northeast Mindanao. The species was first recorded in Agusan Valley (Taylor 1922a; Uetz et al. 2025) and has been reported with increasing frequency in recent years (Taylor 1920a; Taylor 1922a; Gaulke and Alcala 2009; Sanguila et al. 2016; Pitogo et al. 2021; Gamalinda et al. 2024; see Table 1).

## Conclusions

The swamp forests of AMWS provide refuge for the survival of diverse and unique biodiversity (Tandang et al. 2014; Sucaldito-Salibad and Nuñeza 2014; Sumilhig et al. 2024). Even though the vicinity of the AMWS is heavily threatened by timber poaching, small-scale mining, heavy metal pollution, sedimentation, land conversion into agriculture, and the presence of invasive alien species (pers. comm. PASu Emmillie Iboña to MBS, 5 December 2018), the terrestrial land vertebrate biodiversity of Agusan Marsh remains an impressive and yet incompletely characterized quantity. As a testament to this fact, we emphasize our continually expanding documentation of Agusan Marsh herpetofauna and climbing species cumulative totals, which also serve as an illustrative example, emphasizing the need and justification for further field-based survey-and-resurvey sampling, in hopes of arriving at a comprehensive faunal inventory, for further appreciating and protecting the biodiversity of the swamp forests of the Mindanao faunal region. On this latter point, we identify the following research objectives, in the form of questions for consideration in future studies:

1. Given the seasonal cycle of flooding in peat swamp and freshwater swamp forests that account for the emergent vegetation in each habitat type (Giesen 2018), what biotic and abiotic factors influence community composition and structure of the biodiversity in each of these habitat types? Which among the habitat types in swamp forests holds the highest species diversity?
2. What could the presence of widespread, range-restricted, co-distributed species (e.g., *Kurixalus appendiculatus*, *Limnonectes leytensis*, and *Emoia ruficauda*) tell us about their ability to adapt and survive in unique habitats of both swamp and terrestrial forests within the Mindanao faunal region (see Gonzalez et al. 2013; Kaatz et al. 2021)?
3. What possible human-mediated dispersal and/or colonization scenarios can be inferred in relation to non-native species occurrences (e.g., *Kaloula pulchra*, *Indosylvirana nicobariensis*) recorded from Agusan Marsh (see also Diesmos et al. 2006; Brown et al. 2012a)?
4. Drivers of biodiversity loss in tropical forested communities in Southeast Asia include climate change and agricultural expansion (Coleman et al. 2019). Considering the perceived increasing agricultural expansion and decreasing wetland types of vegetation in the protected areas (peat swamps and swamps in this study) of Agusan Marsh (Makinano-Santillan and Santillan 2021), how many and which of the herpetological species recorded by us are vulnerable to climate change (Alcala et al. 2012) and are at extinction risk (see Chapple et al. 2021)?
5. Agriculture (rice farming) and fishing are the major sources of economic and food sustenance for Agusan Marsh communities (Tomas et al. 2011). In this study, we documented invasive alien amphibian species (*Rhinella marina*, *Kaloula pulchra*, *Hoplobatrachus rugulosus*, *Indosylvirana nicobariensis*) co-occurring with native amphibian species (*F*e *jervarya vittigera*, *Occidozyga laevis*) from the rice fields and adjacent areas within sampling sites. Our informal interactions with the local communities at these study sites revealed that they consume amphibians (*R. marina*, *F. vittigera*). Given these points, it will be a worthwhile endeavor to explore the dynamics of human-wildlife interactions in Agusan Marsh using the study model suggested by Propper et al. (2020).
6. How can field-based biodiversity datasets be leveraged by the Office of the Protected Area Superintendent of Agusan Marsh Wildlife Sanctuary (PASu-AMWS; now Protected Area Management Office, PAMO) and local stakeholders in support of policy, practice-based intervention, and overall conservation action, to strengthen and sustain the protected areas of Agusan Marsh (see Smith et al. 2018; García-Bañuelos et al. 2019)?

## Supplementary Material

XML Treatment for Rhinella marina

XML Treatment for Platymantis corrugatus

XML Treatment for Platymantis guentheri

XML Treatment for Fejervarya vittigera

XML Treatment for Hoplobatrachus rugulosus

XML Treatment for Limnonectes leytensis

XML Treatment for Kalophrynus sinensis

XML Treatment for Kaloula picta

XML Treatment for Kaloula pulchra

XML Treatment for Oreophryne anulata

XML Treatment for Indosylvirana nicobariensis

XML Treatment for Kurixalus appendiculatus

XML Treatment for Philautus acutirostris

XML Treatment for Philautus leitensis

XML Treatment for Polypedates leucomystax

XML Treatment for Nyctixalus spinosus

XML Treatment for Draco bimaculatus

XML Treatment for Gonocephalus semperi

XML Treatment for Hydrosaurus pustulatus

XML Treatment for Cyrtodactylus annulatus

XML Treatment for Gehyra mutilata

XML Treatment for Hemidactylus frenatus

XML Treatment for Hemiphyllodactylus typus

XML Treatment for Lepidodactylus aureolineatus

XML Treatment for Brachymeles orientalis

XML Treatment for Emoia ruficauda

XML Treatment for Eutropis caraga

XML Treatment for Eutropis lapulapu

XML Treatment for Eutropis multifasciata

XML Treatment for Lamprolepis smaragdina

XML Treatment for Lipinia pulchella

XML Treatment for Lipinia quadrivittata

XML Treatment for Lipinia semperi

XML Treatment for Parvoscincus steerei

XML Treatment for Pinoyscincus jagori

XML Treatment for Sphenomorphus fasciatus

XML Treatment for Varanus cumingi

XML Treatment for Boiga dendrophila latifasciata

XML Treatment for Chrysopelea paradisi

XML Treatment for Coelognathus erythrurus erythrurus

XML Treatment for Dendrelaphis marenae

XML Treatment for Dendrelaphis philippinensis

XML Treatment for Oxyrhabdium modestum

XML Treatment for Naja samarensis

XML Treatment for Rhabdophis lineatus

XML Treatment for Psammodynastes pulverulentus

XML Treatment for Aplopeltura boa

XML Treatment for Tropidolaemus subannulatus

XML Treatment for Cuora philippinensis

## Additional information

### Use of AI

No use of AI was reported.

### Author ORCIDs

Marites B. Sanguila https://orcid.org/0000-0002-7840-3112

Ana Cristina O. Labaja https://orcid.org/0009-0009-2009-5573

Justin M. Bernstein https://orcid.org/0000-0002-5249-3340

Rafe M. Brown https://orcid.org/0000-0001-5338-0658

### Data availability

The data underpinning the analysis reported in this paper are deposited at GBIF, Global Biodiversity Information Facility, and are available at https://doi.org/10.15468/t9a5uj.
